# Chromate-Free Corrosion Protection Strategies for Magnesium Alloys—A Review: Part II—PEO and Anodizing

**DOI:** 10.3390/ma15238515

**Published:** 2022-11-29

**Authors:** Ewa Wierzbicka, Bahram Vaghefinazari, Marta Mohedano, Peter Visser, Ralf Posner, Carsten Blawert, Mikhail Zheludkevich, Sviatlana Lamaka, Endzhe Matykina, Raúl Arrabal

**Affiliations:** 1 Departamento de Ingeniería Química y de Materiales, Facultad de Ciencias Químicas, Universidad Complutense de Madrid, 28040 Madrid, Spain; 2 Department of Functional Materials and Hydrogen Technology, Faculty of Advanced Technologies and Chemistry, Military University of Technology, Kaliskiego Street 2, 00-908 Warsaw, Poland; 3 Institute of Surface Science, Helmholtz-Zentrum Hereon, 21502 Geesthacht, Germany; 4 AkzoNobel, 2171 EN Sassenheim, The Netherlands; 5 Henkel AG & Co. KGaA, 40191 Düsseldorf, Germany

**Keywords:** magnesium, coating, micro-arc oxidation (MAO), Cr(VI)-based coatings

## Abstract

Although hexavalent chromium-based protection systems are effective and their long-term performance is well understood, they can no longer be used due to their proven Cr(VI) toxicity and carcinogenic effect. The search for alternative protection technologies for Mg alloys has been going on for at least a couple of decades. However, surface treatment systems with equivalent efficacies to that of Cr(VI)-based ones have only begun to emerge much more recently. It is still proving challenging to find sufficiently protective replacements for Cr(VI) that do not give rise to safety concerns related to corrosion, especially in terms of fulfilling the requirements of the transportation industry. Additionally, in overcoming these obstacles, the advantages of newly introduced technologies have to include not only health safety but also need to be balanced against their added cost, as well as being environmentally friendly and simple to implement and maintain. Anodizing, especially when carried out above the breakdown potential (technology known as Plasma Electrolytic Oxidation (PEO)) is an electrochemical oxidation process which has been recognized as one of the most effective methods to significantly improve the corrosion resistance of Mg and its alloys by forming a protective ceramic-like layer on their surface that isolates the base material from aggressive environmental agents. Part II of this review summarizes developments in and future outlooks for Mg anodizing, including traditional chromium-based processes and newly developed chromium-free alternatives, such as PEO technology and the use of organic electrolytes. This work provides an overview of processing parameters such as electrolyte composition and additives, voltage/current regimes, and post-treatment sealing strategies that influence the corrosion performance of the coatings. This large variability of the fabrication conditions makes it possible to obtain Cr-free products that meet the industrial requirements for performance, as expected from traditional Cr-based technologies.

## 1. Foreword

Magnesium is a lightweight and versatile material that has become increasingly important as a structural material in light of global warming and the urgent need for weight reduction. It is able to provide sustainable and affective green corrosion protection solutions in transport, airspace, electronics, and consumer goods sectors. Due to its highly negative standard electrode potential (E° = −2.37 V_SHE_), Mg is susceptible to atmospheric corrosion if left unprotected [1,2]. This review is motivated by the search for an effective alternative to Cr-based surface treatments for Mg alloys that has been going on for the last two decades, spurred by constantly tightening environment-concerned regulations worldwide.

Anodizing, an electrochemical oxidation process, is an effective method to improve the corrosion resistance of Mg and its alloys by forming a protective ceramic-like layer on their surface. Anodizing is commonly used for Al and Ti alloys, but less so for Mg alloys. Cheaper and technologically easier conversion treatment processes are typically preferred for Mg in engineering applications [3]. For details on advances in chromate-free conversion coatings for Mg alloys in engineering applications, the reader is referred to of the present review [4].

Technically, anodic films are also a conversion type of coating, as their composition comprises elements of the substrate and the electrolyte [5]. The anodic films on Mg alloys, formed in alkaline or acidic (less common) electrolytes, are usually crystalline and porous, with a typical thickness of 5–50 µm. The films typically comprise a barrier layer that isolates the base material from aggressive environmental agents and is characterized by good adhesion to the substrate and considerably greater corrosion protection than that of conversion films. The latter is also because the properties of anodic films can be tailored by altering a variety of factors, such as the voltage/current regime, electrolyte composition, sealing, anodizing cell geometry, etc. This variability of the fabrication conditions makes it possible to obtain products which may be easily adapted to industrial requirements. Large-scale production tends to search for efficient, safe, easy, and fast anti-corrosion treatment procedures with minimal production and operation costs.

The present work forms Part II of our review and summarizes the present and future outlooks for corrosion protection strategies for Mg-based anodizing, including traditional chromium-based processes and newly developed chromium-free alternatives, such as plasma electrolytic oxidation (PEO) technology and anodizing in organic electrolytes. It should be mentioned that although a vast body of work exists on PEO technology as applied to Mg alloys for biomedical applications (which, by necessity, are free of toxic elements), this is outside of the scope of this review; the reader can consult the following references for more information [6,7,8,9]. Rather, the present review pinpoints recent developments in anodic treatment systems for Mg alloys that have been shown to be capable of meeting industrial requirements based on the performance associated with traditional Cr-based technologies.

## 2. Evolution of Anodic Treatments for Mg Alloys

### 2.1. Fundamentals of Anodizing Below and Above the Breakdown Potential

**Figure 1:**
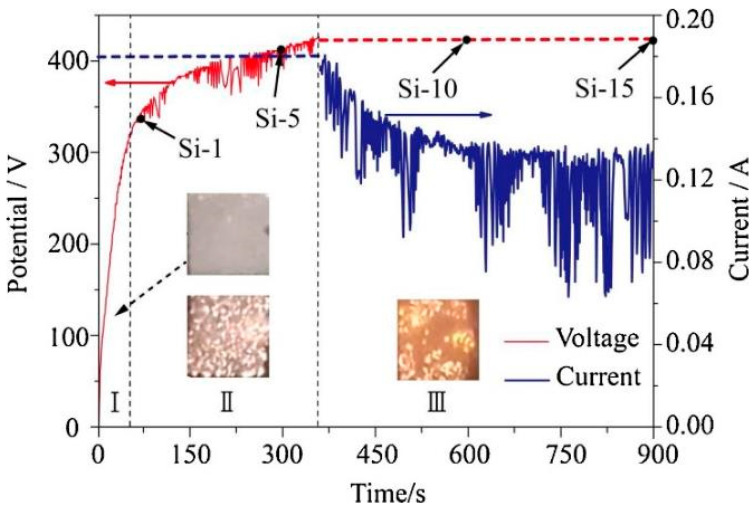
Voltage and current vs. time plots and evolution of discharge sparks. Reprinted from [11] with permission from Elsevier.

Anodizing can be carried out under Direct Current (DC), Alternating Current (AC), unipolar pulsed, and bipolar pulsed conditions with current, voltage, or power input control. In a simple DC mode, potentiostatic or galvanostatic regimes are used, which lead to different morphologies of the resulting anodic films [10]. A constant voltage regime causes a systematic current drop with processing time due to the growth of the dielectric oxide layer and the increase of its resistance. Under current limitations, the increasing resistance of the growing barrier layer causes the voltage to rise with processing time. Figure 1 shows an example of the voltage and current variations during PEO, an advanced form of anodizing. In the initial stage, the process is under constant current control, but once the maximum set voltage is reached, the current decays.

**Figure 2:**
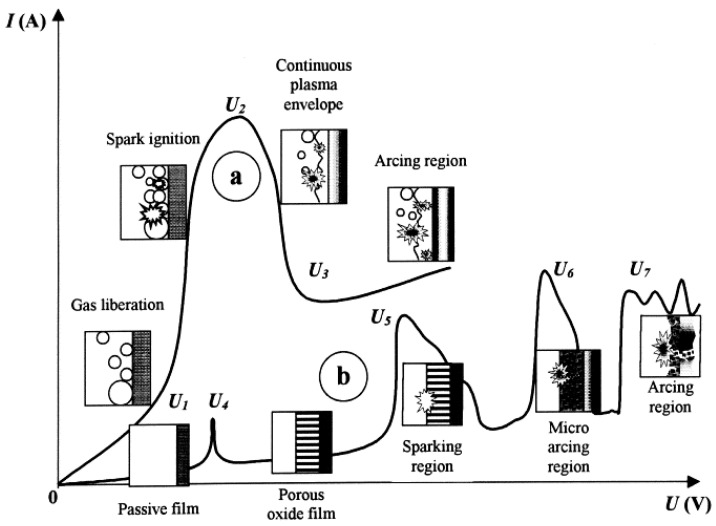
Schematic of current–voltage diagram with the corresponding metal oxide film formation steps during plasma electrolytic oxidation treatment (**a**) in the near-electrode area and (**b**) in the dielectric film on the electrode surface. Reprinted from [10] with permission from Elsevier.

Voltage is a crucial factor that determines the final characteristics of the anodic film. There is a certain potential threshold, i.e., a so-called dielectric breakdown potential, above which the mechanism of the electrochemical oxidation process changes (Figure 2).

Below the breakdown potential, the growth of the oxide coating is relatively slow, and the obtained structure is mostly amorphous. In the case of Mg and its alloys, it is typically composed of hydrated magnesium oxide with a thin barrier layer adjacent to the substrate and an outer finely porous layer, as confirmed by AFM [12] and TEM [13] of the coating cross-sections.

The effect of the electrolyte on the final coating composition in the case of pre-breakdown anodizing is relatively small (the incorporated electrolyte species migrate inward at a constant rate in the electric field). This type of anodic film is not as mechanically and thermodynamically stable, like the aluminum oxide formed during the anodizing of aluminum alloys. The value of the Pilling-Bedworth ratio (molar V_ox_/V_m_) for a MgO/Mg system is only 0.81, and therefore, such oxide films develop cracks [14]. According to the Pourbaix diagram, MgO is stable above pH 10.5, which is out of the typical pH range found in service conditions. This is also the reason why strong acids such as sulfuric and phosphoric acids, typically used in the anodization of Al alloys, cannot be used for anodic treatments of Mg alloys.

The first attempts to anodize Mg were most likely carried out around 1920, with the first patents being filed in 1923 [15,16]. However, the majority of work was done between 1930 and 1950, with the appearance of processes such as AMC K, DOW 9, DOW 13, Framalit (all chromate based), Seomag-W, Seomag G, Elomag, AMC R, DOW 12, DOW 14, DOW 17, CVAC (all alkaline based), Flussal (fluoride based), and Manodyz (silicate based) [17], some of which will be further discussed in Section 2.2.

**Figure 3:**
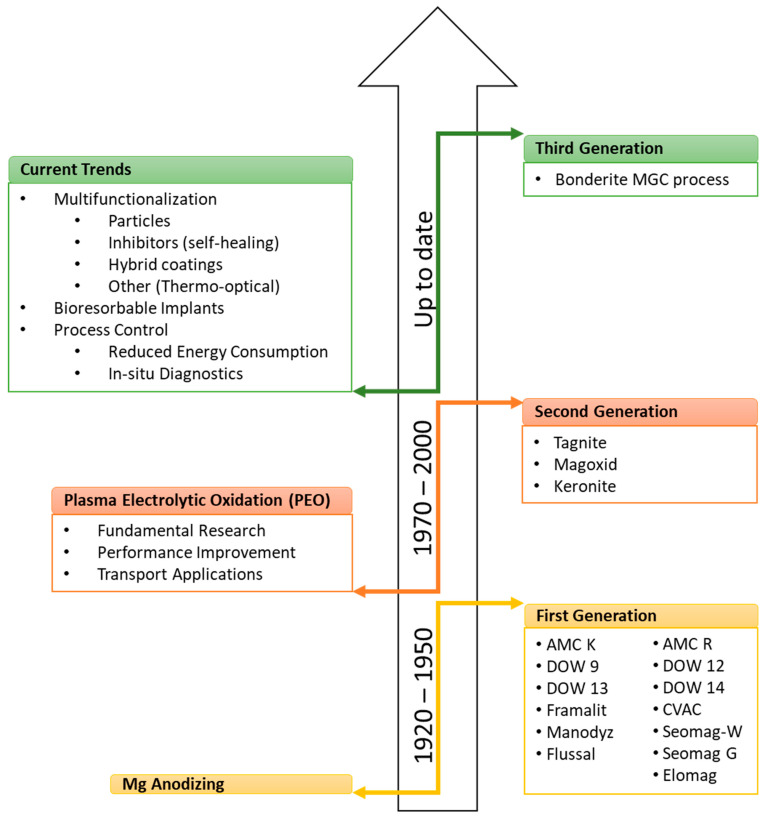
Simplified timeline for the development of PEO coatings on Mg alloys.

Anodizing above the breakdown potential is much more common for Mg alloys, although it has only recently been used on a larger scale. The process is known as plasma electrolytic oxidation (PEO) or micro-arc oxidation (MAO). The beginning of the modern age of fundamental and applied studies of PEO was marked by the works of Brown et al. in the US [18] and Markov et al. in Russia [19] in the 1970s. Our understanding of the process was further developed in the 1980s and 1990s through the work of Fyedorov et al. [20], Kurze et al. [21,22], Snezhko et al. [23,24], and Gordienko et al. [25,26], and the first commercial PEO processes were developed for Al and Mg (Tagnite [27], Magoxide [28], Keronite [29]). An influential review of PEO processes and technology for surface engineering was published by Yerokhin et al. in 1999 [10]. The achievements of the last decade are mainly associated with developments in PEO process diagnostic methods [30,31,32,33,34,35,36], coating multifunctionalization [37,38,39,40,41], and energy consumption reduction [42,43] (Figure 3). Third generation processes aim at the development of electrolytes which are universally suitable for different types of light alloys (Al, Ti, and Mg) and which offer multifunctional performance (e.g., corrosion resistance, abrasion resistance, thermal resistance, final finish). This can be achieved through the use of additives (e.g., particles) and process control.

**Figure 4:**
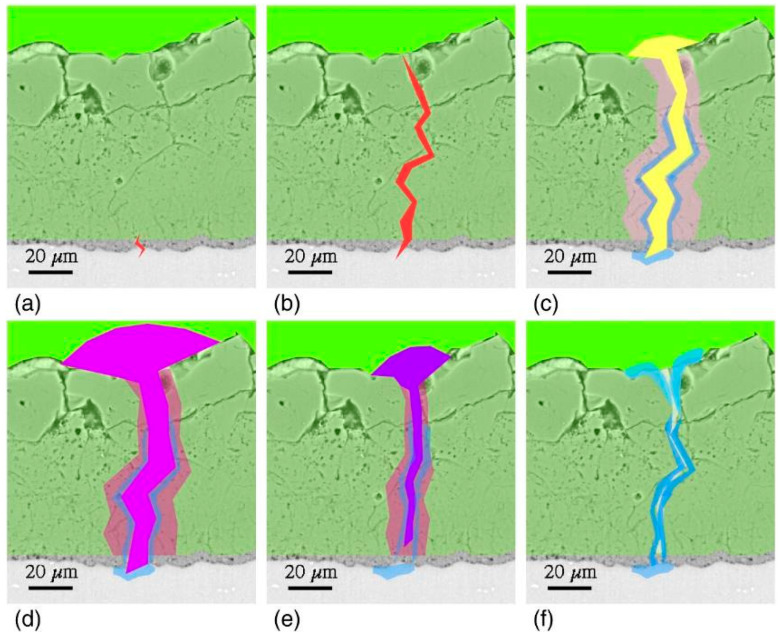
Schematic depiction of the sequence of events during a single discharge, showing: (**a**) initial electrical breakdown, (**b**) the development of the plasma channel through the coating thickness, (**c**) initial bubble growth and formation of oxide in the plasma, (**d**) bubble expansion and heating of the region around the discharge, (**e**) shrinkage and cooling as the plasma resistance rises, causing the current to fall, and (**f**) final quenching and the expulsion of some liquefied oxide from the channel [47].

The formation mechanism of ceramic-like PEO coatings is very complex due to the simultaneous occurrence of several electrochemical, plasma-chemical, and thermal-chemical reactions [10]. Various models have been proposed to describe it [44,45], but the most feasible seems to be the model of oxide film dielectric breakdown [46] (Figure 4).

**Figure 5:**
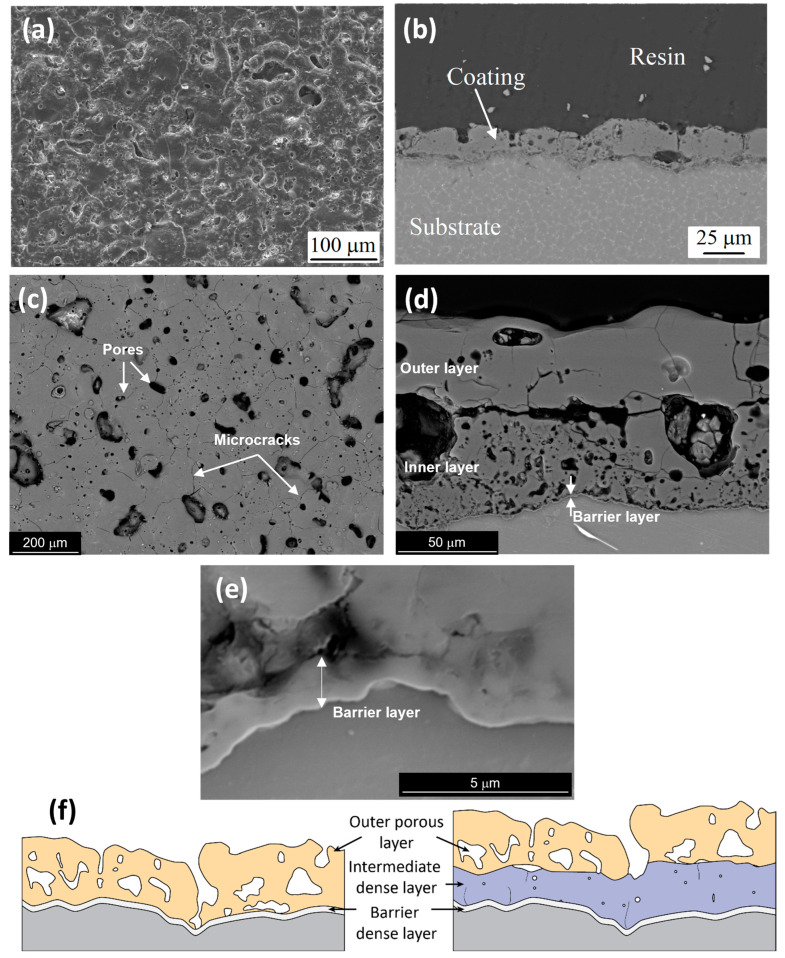
Scanning electron micrographs of two-layer (**a**,**b**) [49] and three-layer (**c**–**e**) [53] PEO coatings on Mg alloys. Schematic diagrams of two-layer and three-layer PEO coatings (**f**). (**a**,**b**) Reprinted from [49], (**c**–**e**) reprinted from [53] with permission from Elsevier.

Under these circumstances, in the initial stage of the process, a thin (100–200 nm) insulating barrier layer is formed, as in conventional anodizing carried out below breakdown potential. In the high voltage region, the electric field across the metal-oxide-gas system is sufficient to generate a dielectric breakdown of the pre-formed oxide that manifests as visible microdischarges (Figure 2). Extremely high localized temperature and plasma pressure, formed from vaporized electrolyte and gas in the discharge channel (~2 × 10^4^ K, 100 MPa) [10,48], cause the ejection of the molten oxide material from the discharge channel onto the coating surface. Then, contact between the liquid phase and a cool electrolyte leads to the very fast solidification of the ejected oxide. This stage occurs with simultaneous pore formation in the middle and outer parts of the coating as a result of the gas passing through the softened oxide material; the coating often contains microcracks caused by thermal stresses and volume changes related to phase transformations (Figure 5a) [49]. The discrete micro-discharges, often occurring in cascades, with the duration of each being in the order of ~10 µs [50,51,52], appear in places where the oxide layer is weakest (e.g., thinnest and/or heterogeneous), causing local thickening of the oxide layer. This sequence of events leads to the synthesis of a three- (sometimes two-) layer coating of uniform thickness. The coating morphology comprises an outer porous section, a compact middle layer, and a thin barrier layer which is well adhered to the magnesium substrate (Figure 5b,c).

The anti-corrosion properties of PEO coatings are mainly attributed to their sub-micrometric barrier layer (Figure 5e) [54,55]. Nevertheless, the middle layer, which often develops under AC regimes, also contributes to the corrosion resistance to a certain extent, since its pores are not penetrating and mostly not interconnected, limiting species diffusion.

**Figure 6:**
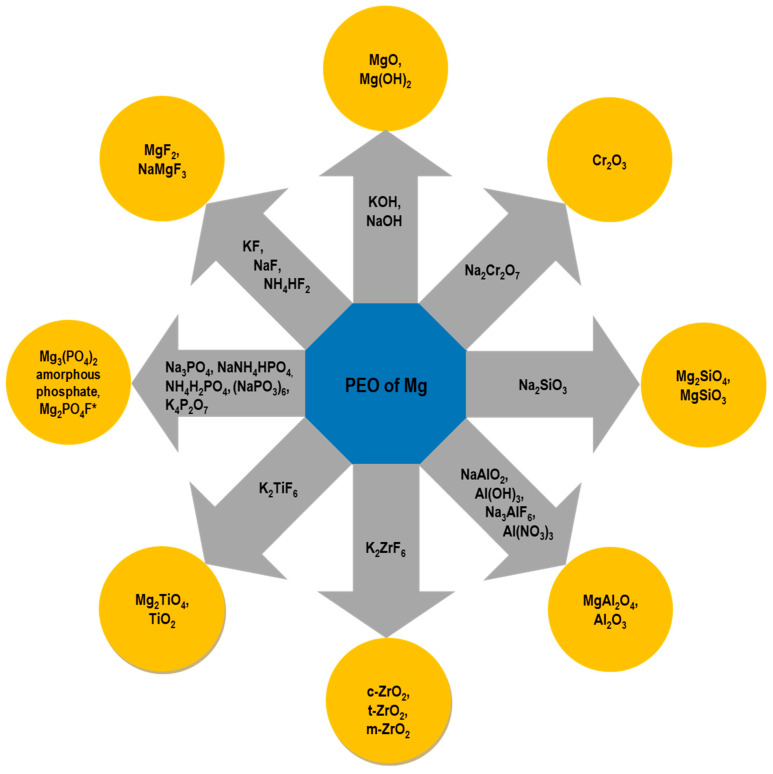
General overview of the most frequently used electrolytes components and phases formed in the PEO process on Mg. * Phase formed in a presence of K_2_TiF_6_.

Anodic films formed above breakdown potential are quite different in terms of their microstructure and morphology compared to those formed below breakdown. The former usually exhibits a crystalline structure, a thicker barrier layer, and a composition that is strongly dependent on the electrolyte constituents, while the latter is easily incorporated during the treatment through short-circuit paths [56]. In alkaline environments, PEO-formed MgO undergoes partial conversion to Mg(OH)_2_, which has a Pilling-Bedworth ratio of 1.77 [57], resulting in greatly improved surface coverage. The ability of PEO processes to incorporate the electrolytes species opens new possibilities for coating design and obtaining the desired properties of surface-treated Mg alloys (Figure 6).

For instance, in the presence of aluminum compounds, a very stable MgAl_2_O_4_ spinel can be formed in the coating in addition to magnesium oxide [58]. This spinel is characterized by excellent chemical, thermal, dielectric, and mechanical features and also has a higher Pilling-Bedworth ratio than MgO (1.3 [59]), which is crucial for providing good corrosion protection. Additionally, PEO coatings on Mg alloys offer advantages like high thickness, fast growth rate, and good paintability, enabled by the relatively rough and porous top part of the coating. Mechanical properties like hardness and wear resistance can also be improved by PEO treatment [6,60]. It is worth noting that, unlike in pre-breakdown anodizing, PEO processing does not require special surface pre-treatment, thus reducing the complexity of the procedure, saving time, and reducing the processing costs, which is especially desirable in industrial applications [61].

Due to the much more desirable properties of the PEO coatings compared with pre-breakdown anodic films on Mg, the last two decades have witnessed a surge of publications on PEO research in China, UK, Russia, Germany, France, Spain, and Turkey [39,49,56,58,62,63,64,65,66,67], associated with the strategic interest of lightweight materials and the need of their protection and functionalization.

### 2.2. Cr-Based Anodic Treatments

**Figure 7:**
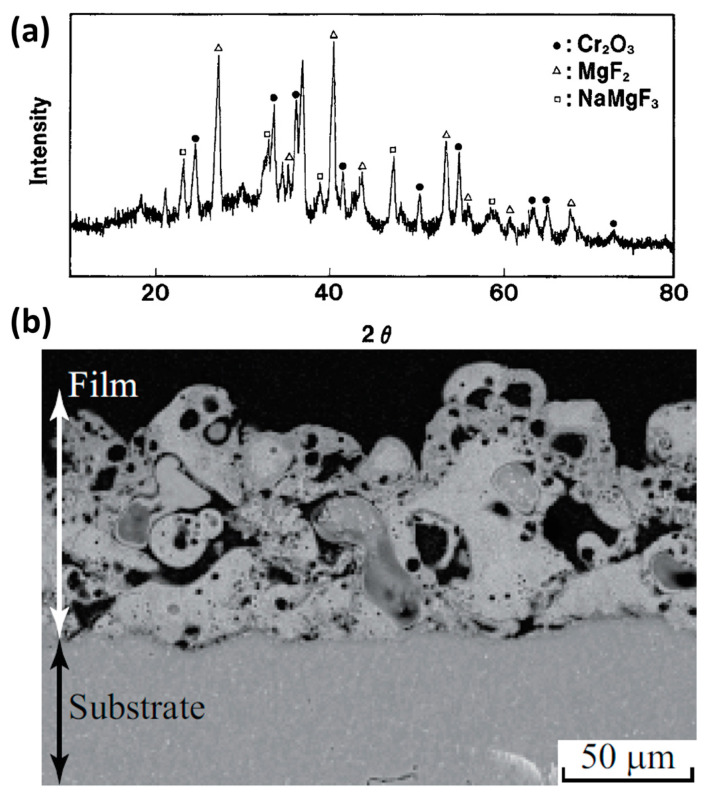
(**a**) X-ray diffraction pattern [71] and (**b**) backscattered electron cross-sectional micrograph of DOW17 coating on AZ91 alloy [72], the latter produced by applying constant current with the voltage rising to 200 V.

As mentioned above, chromium-based protective coatings have been used for a long time. The first satisfactory method of Mg anodic treatment was developed in 1937, and it was Cr-based. The electrolyte was composed of 10% sodium dichromate (Na_2_Cr_2_O_7_) and 2–5% monosodium phosphate (NaH_2_PO_4_). The process was carried out at a constant current of 0.5–1 A m^−2^, 50 °C, for 30–60 min. After painting, the anodic coating exhibited better anti-corrosion properties than a traditional CC obtained by pickling in highly concentrated Cr-based solution (180 g/L of Na_2_Cr_2_O_7_·2H_2_O with 190 mL/L of HNO_3_) and painting [68]. One of the oldest commercial anodizing processes for Mg, DOW17 (1942), which is still commonly used in industrial treatments, also owes its anti-corrosion properties to the presence of Na_2_Cr_2_O_7_ in the electrolyte, with the other components of the concentrated electrolyte being NH_4_HF_2_ and H_3_PO_4_ (pH~5) [69,70]. The DOW17 procedure produces 5–75 µm-thick crystalline coatings containing MgF_2_, NaMgF_3_, Mg*_x_*_+*y*/2_O*_x_*(OH)*_y_*, with some small amounts of Cr_2_O_3_ (Figure 7a), by applying AC or DC at a voltage below 100 V and at a temperature above 70 °C [60,69,71].

An example of the DOW17 coating structure is presented in Figure 7b. Note that its morphology is strikingly similar to that of PEO coatings. As can be seen, the obtained coating is quite porous and irregular and has significant cracks; nevertheless, the process has been widely used because, for a very long time, there was no better alternative.

Surprisingly, despite the search for environmentally friendly alternatives, Cr (VI)-containing processes are still being developed. For instance, recently, DC anodizing of a Mg-Li alloy was reported in a mixture of K_2_Cr_2_O_7_ and (NH_4_)_2_SO_4_ (pH 5.5, 24 °C, 60 min) [70]. The corrosion-resistant coating was obtained due to reactions (1)–(2):

Mg^0^→Mg^2+^ + 2^e−^ (1)

2Mg^2+^ + 2Cr_2_O_7_^2−^ + 6OH^−^/3SO_4_^2−^ → 2MgCrO_4_ + 2Cr(OH)_3_/Cr_2_(SO_4_)_3_ + 30_2_ + 6e^−^ (2)

In the presence of chromate, the protective coating is formed by a chemical reaction between the electrochemically oxidized Mg and hexavalent chromium, with the latter being partially reduced to the trivalent state. The excellent corrosion performance of anodic and conversion chromium-based coatings is related to several factors. Studies on CCC have shown that after drying samples treated in Cr(VI) solution, dehydration of chromium hydroxide occurs, leading to the formation of amphoteric Cr_2_O_3_ [73]. Cr_2_O_3_ is almost insoluble in water, slightly soluble in acids and alkalis [74], and is characterized by a corundum structure and high hardness (almost 9 on the Mohs scale). Moreover, the presence of trapped hexavalent chromium in the form of either salts or oxides is responsible for the self-healing ability of the coating during corrosion [75]. Additionally, some studies have suggested that the presence of polar oxo-Cr(VI) anions prevents the adsorption of de-passivating anions like chlorides [76].

### 2.3. Cr-Free Anodic Treatments: State-of-the-Art Technologies and Patents

Since Cr(VI) is highly toxic and carcinogenic, Cr-based industrial processes must be replaced by new, environmentally-friendly technologies. Further, there is an unceasing need to improve the functional properties of coatings and to reduce fabrication costs, which both affect the economic sustainability of Mg-based components. The majority of the alternative anodizing technologies adopt safe, alkaline-based and chromate-free electrolytes. The optimal electrolyte compositions and process parameters that provide the best coating properties are being intensively researched.

One of the first commercial treatments which departed from the use of chromates was HAE (1955) [77]. The highly alkaline electrolyte (pH~14), composed of KOH, Al(OH)_3_, K_2_F_2_, Na_3_PO_4_, and K_2_MnO_4_, operates at 20–30 °C. The treatment is carried out under an AC regime at 1.5–2.5 A dm^−2^ and, in most cases, the final voltage does not exceed 125 V. The coating thickness ranges between 5–75 µm and is determined by the cut-off voltage limit (60 V for thinnest coatings). The processing time varies between 7 and 60 min for thin and thick coatings, respectively [60]. The quality of the obtained coatings is reported to surpass that of DOW17 [78].

**Figure 8:**
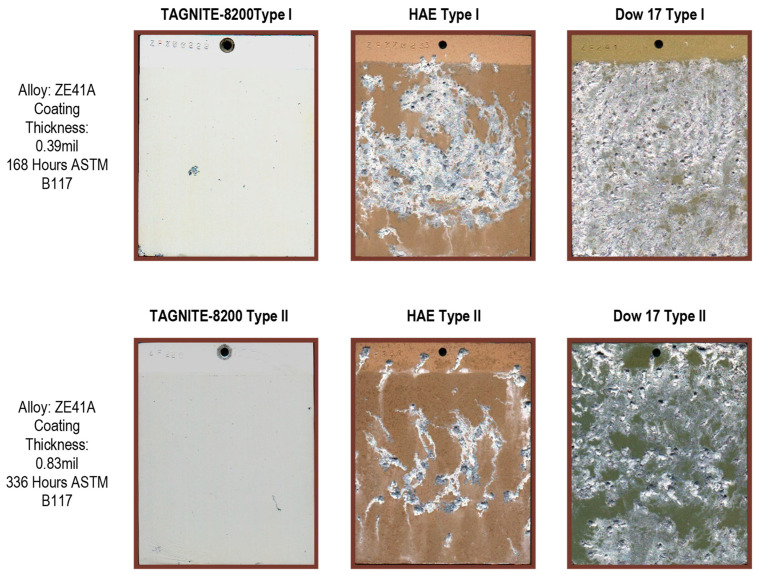
Magnesium test plates after TAGNITE-8200, HAE, and Dow 17 treatments and exposure to salt spray (ASTM B117) for 168 (Types I) and 336 (Types II) hours. Adapted from [79].

Modern commercial developments in Mg anodizing technology offer anti-corrosion properties that are at least comparable to or better than older, acidic chromate-based processes like DOW17 or fluoride-based HAE (Figure 8).

The commercial anodization Anomag (Magnesium Technology) process, as well as PEO processes like Keronite (Keronite), and Magoxid-Coat (Aalberts Surface Technologies), use Cr-free dilute alkaline solutions. The treatment conditions and anti-corrosion characteristics of popular commercial procedures, as well as selected recent related studies, are presented in Table 1.

**Figure 9:**
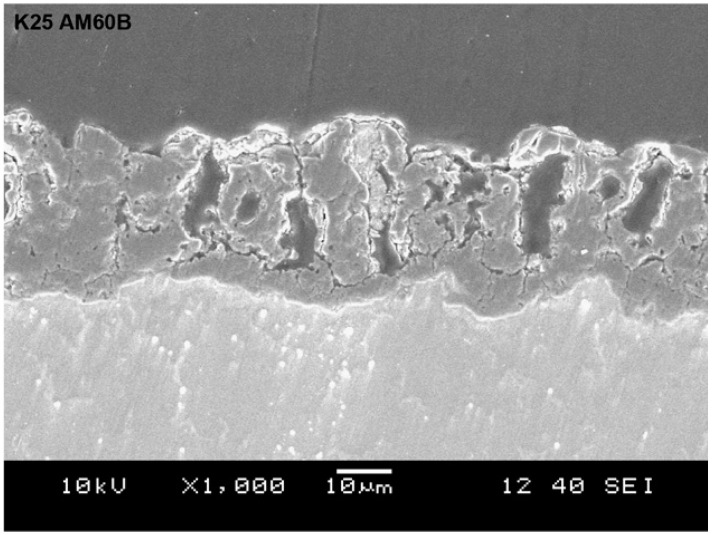
Cross-section SEM image of a 25 µm-thick Keronite coating on die cast AZ91D. Reprinted from [86] with permission from Elsevier.

It can be seen that commercial processes are based on silicate, phosphate, and sometimes, fluoride compounds. The coating corrosion resistance properties are often evaluated in terms of their performance in neutral salt spray tests (NSST); unfortunately, different coating thicknesses make the comparison of different commercial processes difficult. However, it is evident that newer commercial treatments, which abandon the use of toxic compounds, show much greater corrosion resistance than older methods like the DOW17 or HAE. A cross-section view of a Keronite coating [86] is shown in Figure 9.

The last two rows in Table 2 present selected research results. They demonstrate that the electrolytes containing compounds like vanadates [84] or borates [76] can provide better anti-corrosion properties compared with HAE and DOW17 treatments, which are the most commonly used commercial techniques. Nevertheless, it must be considered that both chemicals are hazardous, toxic, and suspected of damaging fertility, similarly to chromates [87,88].

It is worth noting that PEO processes typically work in a high voltage range and therefore are characterized by relatively high energy consumption. Expensive power supplies with high energy output and specially designed output waveforms are often required. These drawbacks limit the application of PEO technology for the production of large parts in the aircraft or automotive industries; this will be discussed further below.

The number of patents of anodizing techniques for Mg alloys has been steadily increasing over the last 20 years (Table 2). As can be seen in the observations, many of these patents focus on the chemistry of the electrolyte and the effect of additives such as particles and organic/inorganic compounds. It is also common to find patents where two-step processes are used to confer additional functionality to the coating.

**Table 2:** PEO patent overview.

Patent or Application No./Title	Observations	Ref.
WO/2017/064185 Corrosion inhibitor composition for magnesium or magnesium alloys	Method Salicylic acid derivatives encapsulated in micro-/nanoparticles as corrosion inhibiting molecules for Mg coatings. Outcome Corrosion protection and improved corrosion inhibiting efficiencies compared to 1,2,4-triazole and benzotriazole as known from US 6,569,264 B1.	[89]
Ru0002614917 Method for protective composite coatings production on magnesium alloy	Method Three steps treatment: PEO coating in silicate-based electrolyte followed by dipping in a tetrafluoroethylene telomer solution in acetone and then heat treatment. Outcome Increased service life and improved corrosion resistance, anti-friction, and hydrophobic properties of the coated material.	[90]
Ru0002543580 Method of obtaining protective coatings on magnesium alloys	Method Four steps treatment: PEO coating in silicate-based electrolyte, dipping in 8-oxyquinoline C_9_H_7_NO solution, followed by boiling in NaOH and posterior heat treatment. Outcome Reduction of corrosion rate, self-healing properties, increased service life in high humidity environment containing Cl.	[91]
US20140318974 Corrosion and erosion-resistant mixed oxide coatings for the protection of chemical and plasma process chamber components	Method Oxide layer formed by PEO with the presence of oxides of secondary elements (not present in the alloy) coming from different sources: (i) a soluble salt of the secondary element(s) in the electrolyte; (ii) an enrichment of the surface of the substrate metal with the secondary element(s) prior to PEO processing; (iii) a suspension of the secondary element(s) or oxide(s) of the secondary element(s) applied to the oxide of the metal after this has been formed by the PEO process. Outcome Corrosion and erosion-resistant mixed oxide coatings.	[92]
DE102011007424 A1 A process for producing a coating on the surface of a substrate based on light metals by plasma electrolytic oxidation	Method PEO in a clay-containing phosphate/silicate electrolyte. Outcome Fabrication of amorphous and glassy oxide surface layer.	[93]
EP1820882 Self-healing layer on nonferrous metals using polyoxometalates	Method Formation of an oxide or metallic layer comprising at least the POMs (e.g., Mo, V, W) and/or a crack-healing agent (e.g., CaO·Al_2_O_3_ or CaO·2Al_2_O_3_). These layers can be deposited/grown on the substrate via conventional anodizing, hard anodizing, PEO, and electroless deposition. Outcome Protective multifunctional layers with self-healing properties.	[94]
WO/2006/007972 Method for producing a hard coating with high corrosion resistance on articles made of anodizable metals or alloys	Method PEO & anodization process in a neutral/alkaline, phosphate-derivatives containing solution. Additional additives: Silicates, H_2_O_2_, alcohol, and either Zr, Ti, or Al-particles. Outcome High hardness and high corrosion resistance coatings.	[95]
US20040238368 Magnesium anodization methods	Method Anodization process in a phosphate alkaline electrolyte containing a sequestering agent to suppress plasma during anodization and a tertiary amine. Outcome -Controlled coating thickness and porosity by choosing various combinations of both current density and time (e.g., high current density for a short time produce a less porous layer). -The addition of a small amount of a phosphonate such as “Dequest” 2066 or 2041 to the anodizing bath allows the anodizing process to proceed with both pulsed waveforms and also DC.	[96]
WO/2003/083181 Process and device for forming ceramic coatings on metals and alloys, and coatings produced by this process	Method PEO (at high frequency pulses) supported by sonic acoustic vibrations for the use of stable hydrosols as electrolytes (for the introduction of fine-disperse particles). Outcome -Controlled micro-discharges during PEO process. -Improved energy efficiency. -Low-porous, 150 µm thick, hard microcrystalline ceramic coatings.	[97]
WO/2003/016596 Magnesium anodization system and methods	Method Anodization process in a phosphate alkaline electrolyte containing a sequestering agent and a tertiary amine. Outcome Process to create oxide layers with sequestering agents in the form of ethylene diamine tetramethylene phosphonic acid and DEQUEST.	[98]
CN106119846 a Method for preparing corrosion resistant and abrasion-resistant coating on surface of magnesium alloy	Method Two-step treatment process: micro-arc oxidation treatment followed by microwave plasma vapor deposition. Outcome Improved corrosion and wear properties.	[99]
WO/2016/010541 Electroceramic coating for magnesium alloys	Method Two steps process: Plasma oxidative deposition in a fluoride-containing solution followed by different organic/inorganic surface finishing. Outcome Improved corrosion resistance.	[100]
WO/2015/008064 High thermal conductivity insulated metal substrates produced by plasma electrolytic oxidation	Method PEO in alkaline solution, specified voltage/current parameters, and pulses. Outcome Improved corrosion protection along with high thermal conductivity achieved on surfaces with high dielectric strength.	[101]
US20090223829 Micro-arc assisted electroless plating methods	Method PEO followed by electroless Ni plating (EN). Outcome Duplex coatings revealed superior corrosion resistance to salt spray testing as compared to the traditional EN coatings.	[102]
US20080248214 Method of forming an oxide coating with dimples on its surface	Method AC, DC, or DC pulse treatments in an alkaline electrolyte. Outcome Wear and corrosion prevention.	[103]
US20070270235 Golf club head and method for making the same	Method Sample degreased by weak alkaline, cleaned, and dried followed by PEO in silicate-phosphate electrolyte at 10–45 °C. Outcome PEO layers developed on golf components.	[104]
EP 1793019 A2 Multivalent electrolytic process for the surface treatment of nonferrous metallic material	Method Anodization process in an alkaline electrolyte (pH 7–10) based on phosphate/ammonia, NaOH/KOH/LiOH. Followed by the coloring stage (dye). Outcome Colored oxide layers.	[105]
Il152307 Oxidising electrolytic method for obtaining a ceramic coating at the surface of a metal	Method Standard PEO process in an alkaline solution (alkali hydroxide + oxyacid salt of an alkali metal). Outcome Specimens with semiconductive properties.	[106]
131996 Method of anodizing magnesium metal and magnesium alloys	Method Anodization of Mg using alkaline electrolytes containing ammonia or an amine and phosphoric acid or a water-soluble phosphate salt. Outcome Details of different processes methodology.	[107]
WO/2003/002776 Method of anodizing of magnesium and magnesium alloys and producing conductive layers on an anodized surface	Method Anodization electrolyte: hydroxylamine, phosphate, nonionic surfactant, alkali hydroxide. Process followed by rendering anodized Mg with an electrolyte containing Ni, pyrophosphate, hypophosphite, and thiocyanate/lead nitrate. Outcome Conductive layers on anodized Mg surface.	[108]
WO/2002/031230 Method for anodizing magnesium and magnesium alloy components or elements	Method and outcome Anodization process using alkaline electrolytes containing phosphates/aluminates.	[109]
WO/1998/042892 Anodizing magnesium and magnesium alloys	Method and outcome Anodization of magnesium or magnesium alloys using an electrolytic solution (preferably derived from phosphoric acid) containing ammonia, amines, or both.	[110]
US5385662 A Method of producing oxide ceramic layers on barrier layer forming metals and articles produced by method	Method and outcome Plasma chemical oxidation of Mg and other metals. Electrolyte: Phosphate, borate, fluoride, stabilizer urea, hexamethylendi(or tetra)amine, glycol/glycerin.	[111]
DE4104847 A1 Production of uniform ceramic layers on metals surfaces by spark discharge- used for metal parts of aluminium, titanium, tantalum, niobium, zirconium, magnesium, and their alloys with large surface areas	Method and outcome Metal parts are immersed in an electrolytic bath (without cathode) and connected to a controllable power source supplying time-dependent, multiphase, periodic current.	[112]
US4976830 A Method of preparing the surfaces of magnesium and magnesium alloys	Method and outcome Mg anodization in electrolyte containing alkali hydroxide, borate/sulfonate, phosphate/fluoride.	[113]
US 3956080 Coated valve metal article formed by spark anodizing	Method and outcome PEO conducted in alkaline electrolyte based on silicates and containing oxyacid of Te or Se.	[114]

## 3. Corrosion of Anodized Mg Alloys

Understanding the influence of the anodic coatings on the mechanisms of corrosion of Mg alloys is crucial for the identification and design of coating characteristics that ensure good protection. The stages leading to corrosion initiation in the presence of anodic coatings are discussed below.

**Figure 10:**
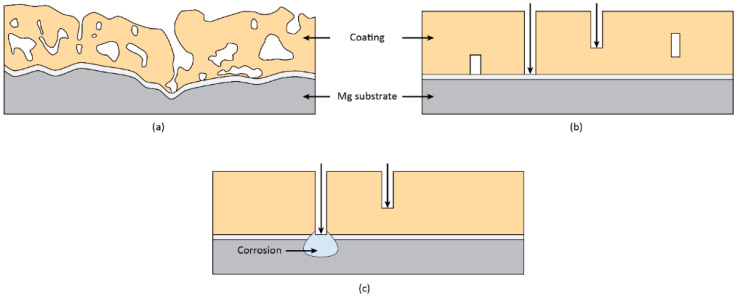
Schematic diagrams of (**a**) PEO coating, (**b**) simplified model of pores, and (**c**) corrosion initiation.

A protective anodic layer separates the alloy from the environment; its main role is to delay the ingress of aggressive species to the substrate [1]. As a consequence, features like big cracks and pores in the coating that enable easy access of the electrolyte to the metal and quick initiation of the corrosion process are undesirable. This is the case for the top porous layer of the coating, which is non-protective. The thick, intermediate layer with discontinuous porosity can considerably delay the corrosion process because the solution cannot easily infiltrate the coating [54,115]. The compact pore-free barrier layer, adjacent to the substrate, is the most protective part of the coating. Defects and partial dissolution of the outer layers, especially when they are amorphous, eventually lead to the penetration of corrosive species down to the barrier layer. The stability of this layer is greatly influenced by the local pH. Low values lead to the fast dissolution of the barrier layer and the corrosion of the substrate, whereas high pH values promote the formation of Mg(OH)_2_, which is voluminous and causes a blocking effect that limits the corrosion rate. This alkalization is particularly facilitated in the limited volume of the pore band that is sometimes present between the barrier layer and the intermediate porous layer [116]. A schematic of the corrosion process is shown in Figure 10.

**Figure 11:**
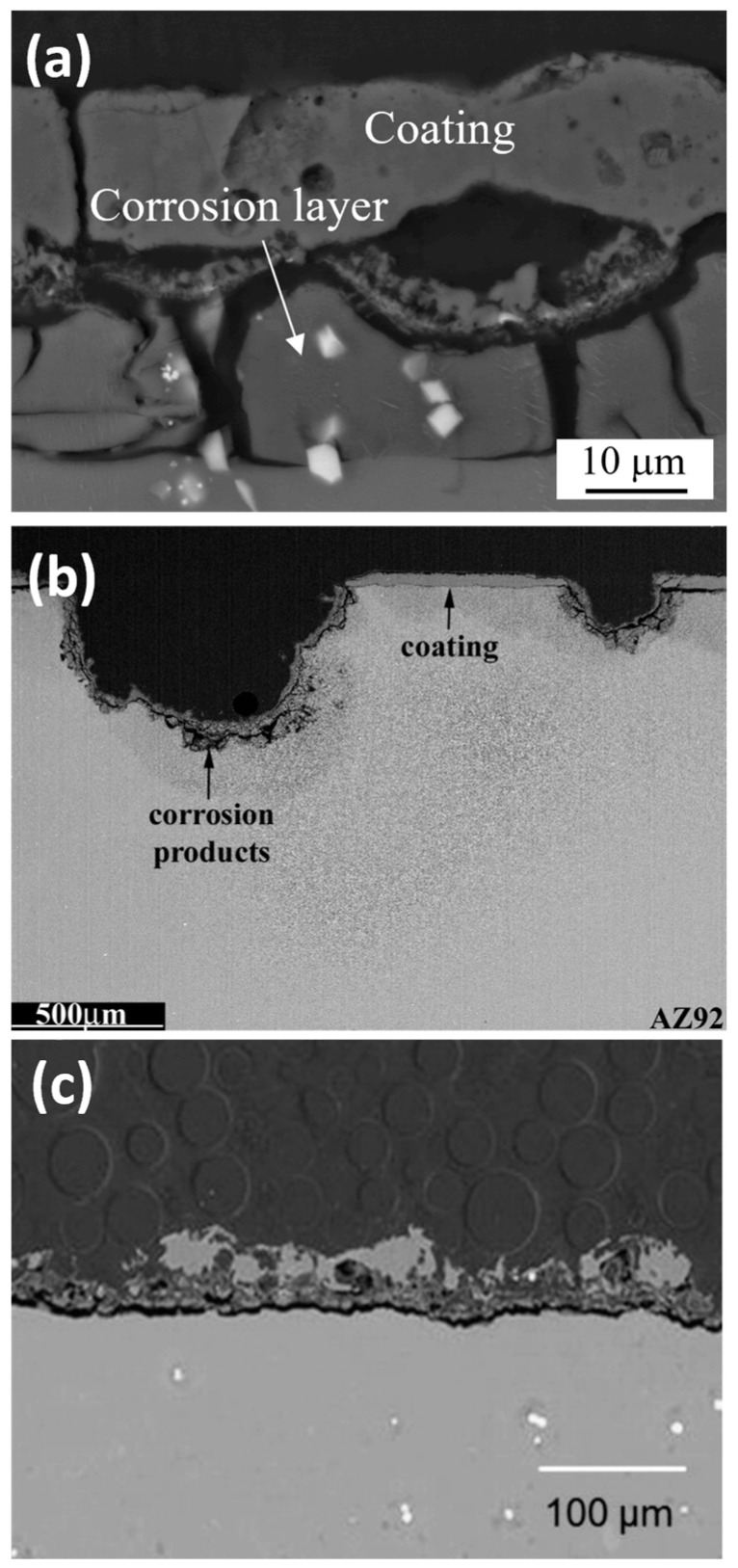
SEM microphotographs showing the types of corrosion morphologies of PEO-coated Mg alloys: (**a**) general undercoating corrosion [49,117], (**b**) localized corrosion [118], and (**c**) coating dissolution [116]. Reprinted (**a**) from [49], (**b**) from [118], (**c**) from [116] with permission from Elsevier.

Typical corrosion morphologies of PEO-coated Mg alloys include general undercoating corrosion [49,117], localized corrosion [118], and coating dissolution [116,119] (Figure 11).

Another aspect to bear in mind is that the influence of the type of Mg alloy only becomes relevant when the PEO coating has failed [54]. Grain boundaries or impurities in the Mg alloy, despite being active corrosion points in the bare substrate, are not so active when covered by a dielectric passive film. Indeed, the probability that continuous pores will be located directly above the active point is small; therefore, the corrosion process is slowed [55]. However, recent research has shown that the second phase segregates in the substrate may also contribute to more extensive discharges and increase the probability of through-going pores there [120]. The impurity level determines how long the coating survives under the same test conditions; this is particularly true for flash-PEO of alloys with strong micro-galvanic coupling [121].

Considering the described mechanism, the corrosion of PEO-coated Mg alloys can be minimized by enhancing of the chemical stability of the coatings and reducing the pore number and size (especially open porosity), which can be realized in several ways during and/or after anodizing. Fine-tuning the electrical input characteristics, the in situ incorporation of corrosion inhibiting species into the coating from the electrolyte, and sealing the pores during post-treatment are examples of current, successful strategies. Further, PEO coatings exhibit very good paintability due to their high roughness; therefore, the application of polymer top-coats significantly improves the corrosion resistance.

## 4. Effect of Energy Input and Electrolyte Composition on Coating Protection Properties

### 4.1. Energy Input

Anodic treatments can be performed using either constant voltage or constant current (DC/AC) regimes or in more advanced unipolar and bipolar pulsed modes. The energy driven into the system feeds several processes: the electrochemical oxidation and Joule heat in the case of conventional anodizing and plasma chemical reactions, thermal oxidation, and electrolyte vaporization in the case of the PEO [122,123,124,125]. As a result, the way the energy is supplied and distributed has a significant impact on the final structure of the coating [126,127,128,129]. For instance, DC constant current conditions sustain intensive, long-lasting, and less mobile micro-discharges which promote the formation of large channels and coating material destruction [130,131], greater pore size, and lower pore population density. Eventually, DC micro-discharges transform into microarcs, accompanied by more intensive gas evolution, the formation of very large size pores, and the thermal cracking of the coating [10].

Typically, anodic treatments of Mg and its alloys use current densities between 0.01–0.1 A cm^−2^. The ranges of final voltages are quite varied. The lowest values can be found below 50 V [132], whereas the highest can be up to 500–600 V [66,133]. In most cases, applied voltages are within the 150–350 V range [60]. Compared to aluminum, the durations of anodic treatments of Mg-based alloys are relatively short. Typically, within 3–15 min, the coating thickness can reach about 4–40 µm, with a growth rate of 0.5–13 µm min^−1^. These thicknesses are not maximal for Mg-based PEO coatings but are optimal for practical applications, especially from an economical and industrial point of view. Optimizing the PEO parameters makes it possible to achieve relatively low energy consumption, for instance, 0.632 kWh m^−2^ µm^−1^ [134]. It was recently shown that so-called flash-plasma electrolytic oxidation coatings (FPEO), with a process duration <90 s and corrosion performance better than that of a commercial Cr(VI)-based coating, consume a very low amount of energy (~1 kWh m^−2^ µm^−1^) [135,136]. In concentrated alkaline electrolytes (>150 g/L dissolved solids) under a DC regime, the energy consumption can be as low as ~0.07 kWh m^−2^ µm^−1^ [137]; however, there is a lack of information on the corrosion behavior of such coatings.

Nowadays, unipolar or bipolar current pulse modes are frequently used in PEO of Mg alloys, as they can decrease the coating porosity and improve homogeneity, and thus, improve the corrosion resistance. Combinations of anodic and cathodic pulses and modifications of the frequency and duty cycle provide a wide range of energy input options that can help to avoid destructive, long-lasting micro-discharges, increase their population density [138], and obtain denser coatings with better corrosion resistance. Some examples of different energy input-related processing parameters and their effect on coating properties are summarized in Table 3.

As can be seen in Table 3, there are many possibilities in terms of modifying the energy parameters, but the final effect on the anti-corrosion properties of the coatings is always a result of all system factors, such as voltage, current density, frequency, and duty cycle, and it is hard to determine the effect of an individual variable. For instance, the influence of the application of uni- or bipolar pulses modes on corrosion resistance is ambiguous, as shown in the contradictory results presented in [144,145,146,148]. Current frequencies tested in the range of 10–2000 Hz exhibit a tendency of corrosion resistance increase with higher frequency values [139,140,142,149]; however, in one case, the opposite trend was observed [143]. Based on the summarized results, it can be concluded that duty cycles should typically be below 40% for both unipolar [139,140,149] and bipolar treatments [141,146], and that higher values are not suitable for the improvement of the anti-corrosion properties of PEO coatings [139]. The coating growth rate in these conditions can be slower than under DC; nevertheless, in well-chosen conditions, it is comparatively fast, usually between 0.77–12.44 µm min^−1^ (Table 3). The influence of system parameters on coating thickness can be arranged from the most to least relevant as follows: final voltage > current density > duty cycle > frequency. The significance of individual parameters on the anti-corrosion properties may be arranged in the following order: final voltage > frequency > duty cycle > current density [140]. Importantly, a literature analysis confirmed that high thickness is not always a guarantee of the best anti-corrosion properties of PEO coatings on Mg alloys.

### 4.2. Electrolyte Composition

The type and concentration of the electrolyte play a crucial role in the morphology, composition, and ultimately, in the corrosion resistance [8,60]. The first and most basic distinction is the pH of the solution. Acidic electrolytes are not preferred for PEO treatment because they completely suppress micro-discharges below pH 3 [150], enhancing the oxide dissolution rate and leading to the formation of porous-type structures with poor corrosion resistance [151,152]. Electrolytes with higher pH values promote the earlier onset of micro-discharges, and the oxide film develops typical PEO three-layered structures.

To ensure an alkaline environment, KOH or NaOH are often chosen as primary components. This also prevents excessive anodic dissolution. With regard to the specific role of K^+^ or Na^+^ ions, it has been shown that the former (as a hydroxide, phosphate, or both) promotes more compact coatings, whereas the latter reduces the pore size in the outer part of the coating [153]. The effects of KOH or NaOH concentrations on coating properties have been intensively investigated. For instance, a three-fold increase of KOH concentration (up to 0.27 M) increased the pH (by ~1 unit) and conductivity (by more than 2.5 times), decreased the breakdown and final voltage values, and increased the compact layer thickness and the MgO/Mg(OH)_2_ ratio, resulting in higher corrosion resistance of coating in salt spray [154]. Similar results were found for a fluoride- and phosphate-based electrolyte containing 1.5 M and 3 M KOH, i.e., better corrosion performance was observed with a more concentrated solution due to the reduced coating porosity [155].

Comparing the results for 5 g/L and 100 g/L NaOH-based electrolytes, the breakdown voltages were 282 V and 82 V, respectively. Furthermore, the coating obtained from the concentrated electrolyte exhibited higher corrosion resistance (two orders of magnitude lower corrosion current and one order of magnitude higher total impedance, |Z|_10mHz_) due to the increased coating thickness [156]. It is evident that due to the effect of decreasing breakdown voltage, the concentration of the electrolytes is a significant factor in minimizing the energy consumption. Sustainable recycling and circular economy routes need to be developed for such electrolytes in order to avoid the increase of the environmental footprint when they need to be disposed of.

Other additives, like phosphates, silicates, fluorides, aluminates, zirconates, permanganates, etc., are used to modify the coating composition and properties. Under the electric field, anions like PO_4_^3−^, SiO_3_^2−^ and F^−^ migrate inward and get incorporated into the coating [157]. The following reactions (3)–(7) are examples describing the different phase formation in the presence of some of these additives:

3Mg^2+^ + PO_4_^3−^ → Mg_3_(PO_4_)_2_ (3)

2 Mg^2+^ + SiO_3_^2−^ + 2OH^−^ → Mg_2_SiO_4_ + H_2_O (4)

Mg^2+^ + SiO_3_^2−^ → MgSiO_3_ (5)

Mg^2+^ + 2F^−^ → MgF_2_ (6)

Mg^2+^ + 2AlO_2_^−^ → MgAl_2_O_4_ (7)

The X-ray diffraction patterns of PEO-treated Mg alloys confirmed the presence of the aforementioned compounds [143,158,159]. These phases can have a great effect on the physico-chemical properties of the coatings; for instance, the following order of corrosion resistance to Cl^−^ attack was identified: amorphous material < MgO < Mg_3_(PO_4_)_2_ < Mg_2_SiO_4_ [116].

The beneficial effect of fluoride-based electrolytes on the corrosion resistance of PEO coatings on Mg alloys is often related to the formation of passivating MgF_2_ phase [160,161], associated with more compact layers, nobler corrosion potential, and reduced susceptibility to pitting [133,160,162,163]. It was also reported that the addition of fluoride to various electrolytes (based on NaAlO_2_, Na_2_SiO_3_, NaAlO_2_ with Na_3_PO_4_, and Na_2_SiO_3_ with Na_3_PO_4_) always caused much quicker growth of the PEO layer, which led to a significant reduction of specific energy consumption [135]. On the other hand, it was shown that similar corrosion resistance can be achieved in more benign, fluoride-free electrolytes, such as aluminate-phosphate [164].

The influence of phosphates, common additives in commercial electrolytes (Anomag CR1 and CR2, Henkel Japan Co., Ltd.), on corrosion properties has been shown [49] and seemed to depend on the various types of phosphates (orthophosphate, pyrophosphate, polyphosphate, hydrophosphates, etc.) and the treatment conditions [165,166,167]. Phosphorus-containing coatings typically favor the formation of amorphous phases, although Mg_3_(PO_4_)_2_ can also be detected [165]. It has been suggested that amorphous phosphate participates in a self-repairing mechanism consisting of its dissolution and subsequent re-deposition as an insoluble crystalline hydrated magnesium phosphate at the locations where the PEO coating fails [168].

The presence of sodium orthophosphate in the electrolyte has been shown to promote the coating growth rate through a more uniform distribution of micro-discharges, avoiding local coating destruction [165]. However, the phosphate concentration must be carefully chosen. According to [164], too low concentrations (<0.21 M) result in non-uniform coatings, whereas concentrations higher than 0.25 M lead to recurrent micro-discharges at specific locations on the surface.

The addition of aluminates, like NaAlO_2_, to the electrolyte has been shown to decrease the number and size of discharge channels [58] and increase the coating thickness [159]. More importantly, the formation of MgAl_2_O_4_ results in enhanced corrosion resistance, which is associated with its chemical stability and a higher Pilling-Bedworth ratio compared with MgO [48,169,170]. It has been proven that for a long immersion time (9 days), the corrosion resistance of the aluminate-containing coating was higher than that of silicate- and phosphate-based electrolytes [171]. Less frequently used aluminum nitrate, Al(NO_3_)_3_, also helps to form a thick and uniform passive layer when used in the 0.15–0.20 M range [164].

Perhaps the most common component of PEO electrolytes is sodium silicate, Na_2_SiO_3_. Compared with equivalent amounts of phosphates and aluminates, it has a greater influence on the electrolyte conductivity, final voltage, and therefore, coating thickness. A corrosion study comparing the effect of phosphate, aluminate, molybdate, and silicate sodium salts revealed that the latter provided the biggest improvement. This was probably due to a higher coating thickness and less open porosity [159].

Frequently, mixtures of the aforementioned chemicals work much better than simple composition electrolytes. The study of the FPEO (>90 s treatment, ~5 µm coatings thickness) obtained from varied electrolytes based on mixtures of KOH with one, two, or three components from NaAlO_2_, Na_2_SiO_3_, Na_3_PO_4_, KF showed the highest performance with the most complex (four elements) compositions (either aluminate or silicate mixtures with potassium hydroxide, phosphate, and fluoride). The best coating achieved superb corrosion protection, significantly outperforming commercial Cr(VI)-based reference samples [135,136].

Sodium borate, Na_2_B_4_O_7_, is usually used in combination with silicate, phosphate, or aluminate salts. Borate ions promote coating growth, since the decomposing B_4_O_7_^2−^ is an additional source of oxygen. The presence of sodium tetraborate in a solution of Na_2_SiO_3_, KOH, NaH_2_PO_4_, and, optionally, KF, improved the coating resistivity by ten fold, although it tended to cause a “coral reef” surface morphology [172]. A Mott–Schottky examination of the electronic properties of the borate- and fluoride-based coatings indicated their lower donor concentration, much more negative flat band potential, and, therefore, higher corrosion resistance. Some other works have also shown the beneficial effect of Na_2_B_4_O_7_ on corrosion resistance and even suggested that it assists in a self-repairing mechanism [158,173].

#### 4.2.1. Organic and Inorganic Soluble Additives

**Figure 12:**
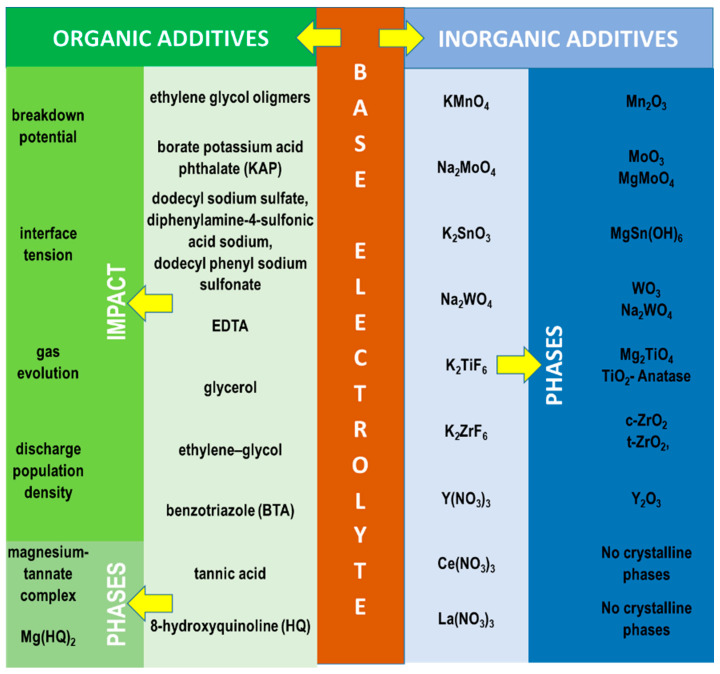
Schematic of soluble organic and inorganic substances added to PEO electrolytes and their effect on the resulting coatings.

The compounds discussed above are the most frequently used constituents of electrolytes in PEO of Mg alloys. However, a lot of attention is currently being paid to the further improvement of the coating properties, which can be achieved by using various types of organic and inorganic additives in the basic electrolyte mixtures (Figure 12).

It should be noted that while the introduction of some soluble inorganic compounds strongly modifies the chemical composition of the coating, it also changes the pH and conductivity of the electrolyte, which influence the breakdown and final voltages and discharge density, resulting in structural modifications to the coatings [173,174,175]. The main criterion behind the choice of an additive salt is its capacity to form highly stable compounds that improve the passivity of the coating [84,132]. For example, elements like Mo (VI) [176] or rare-earth Ce(III) and La(III) [174] have the potential to provide a self-healing capacity of the coating under corrosion.

**Figure 13:**
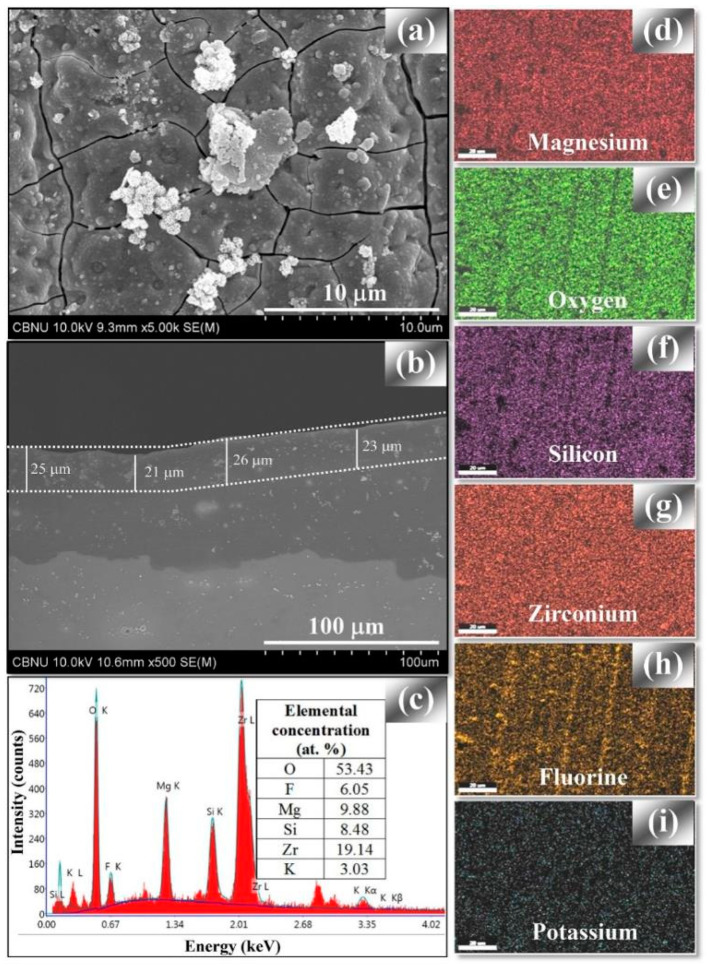
(**a**) Surface morphology; (**b**) cross sectional morphology; (**c**) EDS spectrum, along with the chemical composition. (**d**–**i**) X-ray elemental mapping of the MAO coating deposited on Mg in two stages, sequentially using the alkaline silicate electrolyte in the first stage, followed by the acidic zirconate electrolyte in the second stage, both at 300 V and for 3 min each stage [180]. Reprinted from [180] with permission from Elsevier.

Titanium [177,178,179] and zirconium compounds [150,180,181,182,183,184] are being actively researched as additives due to their very high stability and pore-sealing effect [177,178,184]. While titanium is typically used in a form of K_2_TiF_6_, various types of zirconium salts have been tested. The best results (denser internal film and a more uniform surface) were yielded by K_2_ZrF_6_ compared with ZrOCl_2_ and Zr(NO_3_)_4_ [150]. The inconvenience of K_2_ZrF_6_ is the hydrolysis of Zr^4+^ ions at pH > 4.0, which leads to the precipitation of Zr(OH)_4_ in the electrolyte and, consequently, non-uniform discharge distribution and low coating quality. This limits the choice of basic components of the electrolytes to those that support relatively low pH, such as NaH_2_PO_4_ [182,183], which, in turn, limits the range of coating phase compositions. Some authors have circumvented this by dual- or two-step anodizing (Figure 13), where PEO of magnesium alloy in the acidic electrolytes [180] is preceded by a short anodic pre-treatment in alkaline electrolyte; this prevents the excessive anodic dissolution of the magnesium alloy and ensures good corrosion protection [180,181].

Organic additives usually do not change the coating composition but can provide some unique effects compared with inorganic compounds. Firstly, they can either decrease or increase the breakdown potential [185,186], with the latter imposing an additional economic burden. Further, they adsorb on the electrode and significantly reduce the anode/electrolyte interface tension [66,187], modulating gas evolution and the discharge population density [185,187]. All these effects can affect the mechanism of the discharge and change the structure of PEO coatings, reduce the surface porosity, inhibit defect development and improve the corrosion resistance [188]. The detailed effects of organic and inorganic additives are presented in Table 4.

The analysis in Table 4 indicates that additives in a mixture of 2–3 basic components (e.g., silicates, fluorides, phosphates) can provide significantly improved corrosion protection; however, the final result is not a direct sum of the effects of the individual constituents. Initial studies of the effect of electrolyte additives carried out by McNeil in 1957 demonstrated a positive influence of tungstate, vanadate, and stannate on corrosion resistance (although it is worth noting that the substrates were pickled in dichromate solution before anodic treatment and, optionally, after treatment). The best results before and after the post-treatment were obtained in the presence of vanadate.

In most of the cases presented in Table 4, the additives led to enhanced corrosion protection compared to additive-free PEO coatings (higher corrosion potential, lower corrosion current, lower tribocorrosion rate) [84,174,175,176,184,185,186,187,191,192,196,197,201]; however, negative results were also reported [132,189]. Often, the additives demonstrated positive effects within a certain concentration limit, beyond which the properties deteriorated [126,176,179,185,192,201,202]. It is also evident that additives in the electrolyte often reduce the coating thickness, without reducing the coating protective properties, which is associated with improved morphological parameters of the coatings, such as compactness, roughness, or pores size [174,175,192,196,203,204].

**Figure 14:**
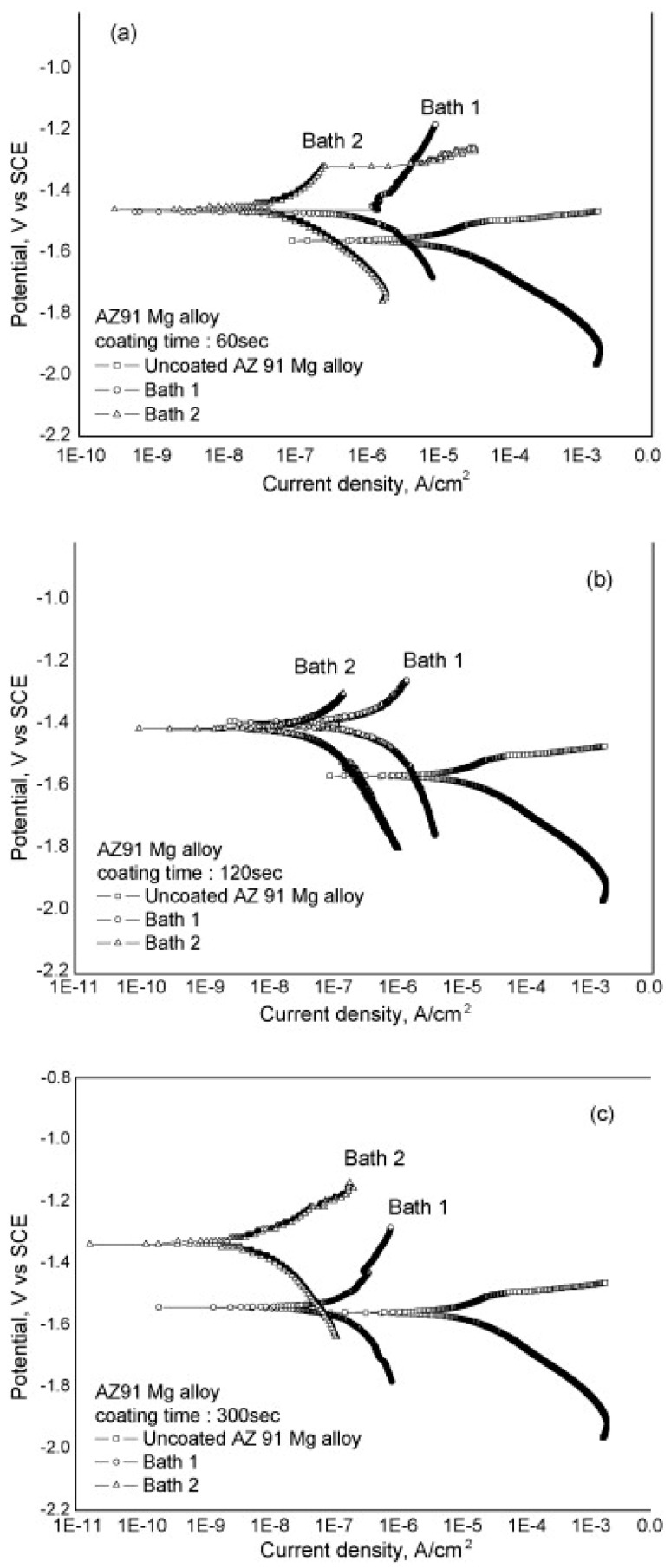
Potentiodynamic polarization curves of coatings formed in electrolyte without (Bath 1) and with KMnO_4_ (Bath 2); (**a**) samples coated for 60 s; (**b**) samples coated for 120 s; (**c**) samples coated for 300 s [175]. Reprinted from [175] with permission from Elsevier.

The reduction of corrosion current by two orders of magnitude compared with the untreated substrate (e.g., down to ~10^−7^ A/cm^2^) is frequently achieved in the presence of an additive. One of the most notable improvements was obtained by the addition of KMnO_4_ (10^−9^ A/cm^2^) (Figure 14) [175] or EDTA (10^−8^ A/cm^2^) into the electrolyte [196].

Recently, a number of works have applied orthogonal experiment designs in order to evaluate the interaction of several electrolyte components, such as K_2_ZrF_6_ and EDTA-Na, with the electrical parameters. Both of these additives are being increasingly used in new PEO coating developments [205,206,207,208]; K_2_ZrF_6_ as a source of Zr, with the aim of forming ZrO_2_ in the coating from particle-free electrolytes, and EDTA-Na as a complexing agent to stabilize cations of interest in the electrolyte [193]. K_2_ZrF_6_ is problematic on its own in terms of the surface passivation and coating growth that occur due to its acidity; however, it can successfully form self-sealing coatings when combined with other additives, such as phytic acid and ammonium fluoride [206].

Organic additives in the electrolyte typically decompose during PEO micro-discharge, leading to excessive gas generation and, as a result, a looser coating microstructure with compromised corrosion resistance. However, additives such as silanes, e.g., 3-aminopropyltrimethoxysilane (APTMS), have been shown to increase the coating thickness and corrosion resistance due to the densification of the coating and the reduction of pore size associated with the formation of Mg-O-Si chemical bonds [199].

The introduction of N and C into the coating via plasma electrolytic nitrocarburizing (PENC) from formamide/NaOH aqueous electrolytes, where Mg acts as a cathode, followed by PEO in an alkaline silicate electrolyte is a new strategy that offers sustainable, high corrosion resistance of a Mg alloy due to the formation of a thick and extremely compact coating [200].

#### 4.2.2. Particle Addition

**Figure 15:**
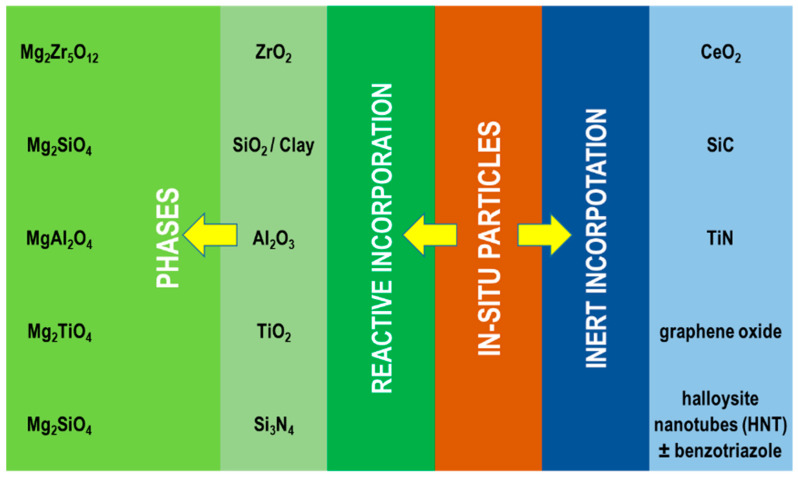
Overview of the insoluble particle types added in situ into the PEO electrolyte and their effect on the formed phases.

New PEO coatings with added functionality are being investigated through the in situ incorporation of insoluble particles in the electrolyte (Figure 15). This approach modifies both the electrochemistry of the PEO process (e.g., conductivity, pH, breakdown voltage, etc.) and the coating characteristics (e.g., porosity, thickness, phase composition, compactness of the layer, etc.). The added particles bring new functionalities to the coating and can be introduced into the coating in an inert manner, without reaction or the formation of new phases, or by reactive or partially reactive incorporation, when a reaction between the particles and the coating matrix takes place [209].

Reactive incorporation includes one of the following: melting, phase transformation, and reaction with electrolyte constituents, substrate, or other phases in the coating. Crucially, the extent of these processes is dependent on many factors, such as the size, melting point, concentration, and the zeta potential of the added particles, the composition of the electrolyte, type of substrate, and energy input from the micro-discharges. Examples of particles that reactively incorporate into PEO coatings on Mg are Al_2_O_3_, ZrO_2_, SiO_2_, and clay particles [209].

The size of the suspended particles should be small enough, i.e., less than 500 nm, to prevent their sedimentation and to enable their easy transport into discharge channels. The zeta potential is an important parameter that characterizes the degree of electrostatic repulsion between the particles in the electrolyte; the more negative its value, the more uniform the dispersion of particles and the more homogeneous their distribution in the coating. Typically, in alkaline solutions, particles are negatively charged, which facilitates their electric field-assisted incorporation into the coating matrix. More information on the stabilization of particles in the electrolytes using organic additives (e.g., urea, sodium dodecyl sulfate) and the mechanisms of particle incorporation into PEO coatings on Mg alloys can be found in [210,211].

Many types of particles can be used to improve the corrosion resistance of PEO coatings on Mg alloys. One of the largest groups includes ceramic oxides such as ZrO_2_ [53,212,213,214,215,216,217], CeO_2_ [53,218,219,220,221,222], Y_2_O_3_ [223], Sb_2_O_3_ [224], TiO_2_ [225,226], Al_2_O_3_ [227,228,229], SiO_2_ [230,231,232,233], and clay [116,234]. Depending on the PEO parameters and thermodynamic stability of these ceramic oxides, they experience either inert [213,226,227,231] or reactive/partly reactive incorporation, resulting in the formation of new stable phases like Mg_2_Zr_5_O_12_, Mg_2_TiO_4_, Mg_2_SiO_4_, or MgAl_2_O_4_ [212,219,220,225,228,230,231]. Improved corrosion performance can also be achieved by the addition of non-oxide particles such as SiC [234,235], TiC, NbC [236], WS_2_ [237], MoS_2_ [238], Si_3_N_4_ [234], and TiN [239,240,241].

Recently, a new range of particles came into focus. For instance, graphene oxide (GO) was successfully introduced into a PEO coating [242,243,244], reducing the number of micropores and improving the corrosion resistance due to the increased tortuosity of the electrolyte species diffusion pathway. Similarly, the addition of graphite [237,245,246], multi-walled carbon nanotubes (CNT) [247,248], or carbon spheres (CS) [249] into the electrolyte produced a coating densifying effect, thereby increasing the corrosion resistance while the CNT oxidized during PEO (new). However, the primary benefits of the incorporation of carbon-based particles are enhanced hardness, wear resistance, and heat dissipation [247,248,249]. Another innovative idea is the in situ incorporation of nanocontainers loaded with corrosion inhibitors (e.g., halloysite or aluminosilicate nanotubes loaded with benzotriazole, molybdate, or vanadate salts or 8-hydroxyquinoline) [250,251,252]. The inhibitor is released when the pH changes during the corrosion process, which confers self-healing properties upon the PEO coating. The effects of particle addition on the coating properties and corrosion resistance are summarized in Table 5.

Based on the results in Table 5, it can be concluded that the incorporation of in situ particles is a very promising way to improve the anti-corrosion properties of PEO coatings. In almost all studies, the corrosion current density decreased compared with the particle-free PEO coatings fabricated under the same conditions. The lowest values were obtained for treatments with the addition of zirconia sol (1.4 × 10^−8^ A/cm^2^) [212], clay (2.3 × 10^−8^ A/cm^2^) [116], and alumina sol (2.6 × 10^−8^ A/cm^2^) [228]. Comparing these values with those shown in Table 4, it is evident that the addition of particles can be more effective than the use of soluble electrolyte additives.

However, the testing period appears to be important. Recently, it was reported that some particle-incorporated coatings provide only a short-term increase in corrosion resistance; longer testing times often reveal the opposite effect, with faster relative degradation [209,234,253]. Additionally, as observed with other electrolyte constituents, particles are only effective up to a certain concentration, usually below 20 g/L, beyond which their positive effect on corrosion resistance declines [239,242]. Their effect on coating thickness is variable and not correlated with corrosion resistance. This notwithstanding, particle-modified coatings are good for additional functionalities, such as, for instance, wear resistance [254].

#### 4.2.3. Organic Electrolytes

Anodizing Mg alloys in non-aqueous electrolytes is an alternative route that has been actively explored in the last ten years [255,256,257,258,259,260,261]. Compared to aqueous anodizing, it avoids water decomposition, decreases Mg dissolution during anodizing, and opens a window for the development of defect-free anodic films. Ethylene glycol, triethanolamine, and glycerol are often used as base electrolytes which may also contain fluoride anions and a small percentage of water. Depending on the source of fluoride ions (HF, NH_4_F, etc.) and the presence of water, barrier type [261] or self-organized nanoporous/nanotubular [260] films can be formed. For instance, ordered oxy-fluoride nanostructures (nanopores and nanotubes) with 70–100 nm pore diameter were formed in a WE43 Mg alloy by anodizing in ethyleneglycol and 0.2 M HF in the potential window between 70 and 120 V [260]. In another study, the effect of water content was explored regarding the transformation from a porous to a compact film morphology. In a solution of ethylene glycol and trimethylamine with 1% water, a porous structure (100 nm pore diameter) was formed, whereas a water content between 10% and 40% led to the formation of a barrier-type film. The latter showed better anticorrosion properties than the nanoporous films in a salt-spray test [259]. FTIR and GDOES results showed the incorporation of C, N, and O in the films and that the amount of incorporated species increased with decreasing water content.

It is worth mentioning that anodizing in organic electrolytes in the presence of fluoride ions helps to increase the efficiency of the process and increases the Pilling-Bedworth ratio up to ~1.7 [261] due to F-enrichment (~0.2 O/F ratio [258]), which is expected to be beneficial for corrosion protection. Efficiencies close to 100% are commonly reported for anodizing using these types of electrolytes (the lower the water content, the higher the current efficiency) [255].

**Figure 16:**
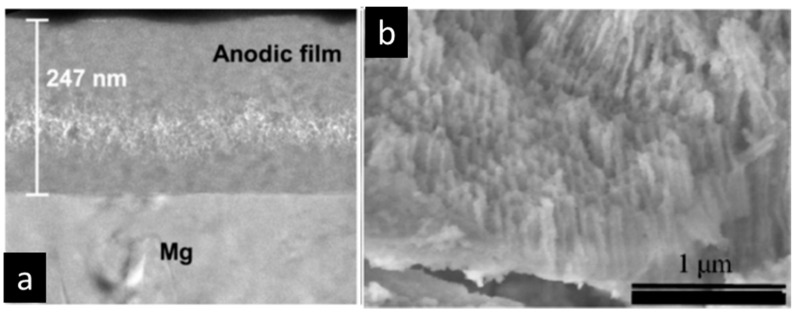
Oxide films formed on Mg alloys in organic electrolytes: (**a**) barrier type [261]; (**b**) nanotubular type [260]. Reprinted (**a**) from [261] and (**b**) from [260] with permission from Elsevier.

Anodizing voltages can be as high as 440 V without dielectric breakdown [257], and the thickness of the coatings ranges from several hundreds of nanometers, in the case of barrier-type films (Figure 16), to several tens of micrometers in nanoporous/nanotubular films (Figure 16b) [256].

**Figure 17:**
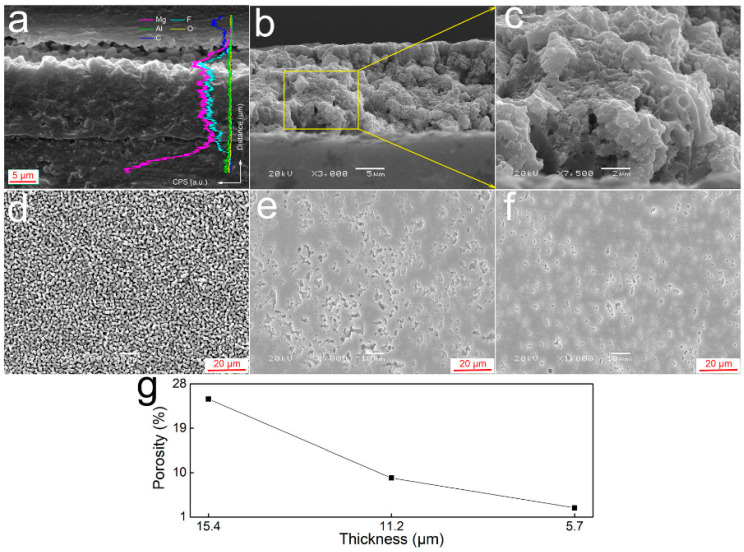
Cross-sectional secondary (**a**–**c**) and planar view (**d**–**f**). Electron micrographs of PEO coating on AZ31B alloy formed in ethylene glycol and NH_4_F electrolyte: (**a**) polished morphology and EDS line-scanning, (**b**) fractured morphology, (**c**) enlarged view of (**b**); morphology of as-coated surface (**d**), the coating polished to 11.2 μm (**e**) and 5.7 μm (**f**); (**g**) porosity statistics [262]. Reprinted from [262] with permission from Taylor & Francis.

Qi et al. recently obtained a ~15 µm-thick PEO coating on AZ31B alloy in ethylene glycol-based electrolyte with 8 wt.% NH_4_F under pulsed bipolar conditions (Figure 17). The coating comprised pure MgF_2_ and had a gradient porosity reducing inward from 24.93% to 2.87%. The coating reduced the corrosion current density of the alloy in neutral and acidic (pH3.0) 3.5%NaCl by approximately two and one orders of magnitude, to ~5 × 10^−7^ A/cm^2^ and to ~3 × 10^−6^ A/cm^2^, respectively [262].

So far, studies on anodizing in organic electrolytes have been carried out using a range of commercial magnesium alloys (e.g., ZE41, WE43, AZ91D, Mg-Zn-RE, etc.). It has been observed that the second phases can be anodized and incorporated into the anodic films, locally affecting the film thickness and composition. However, there is a severe lack of applied and systematic studies of the film properties, energy efficiency, and corrosion behavior.

## 5. Post-Treatment

Sealing the pores provides long-term corrosion protection and can be achieved via different surface post-treatments, including immersion in solutions with inorganic and/or organic compounds [263,264,265], the application of sol-gels [266,267,268,269], electrophoretic deposition [270,271], electrodeposition [272], thermosetting polymers [273,274], and other top-coats [275,276,277]. These post-treatments can also be used with the aim of adding functionality to the surface, e.g., hydrophobicity, aesthetics, etc. [61].

Early approaches to seal the pores included immersion in hot KH_2_PO_4_ or Na_2_SiO_3_ solutions, with higher temperatures and longer treatment times resulting in enhanced corrosion performance [86,278]. The deposition of rare earth and molybdenum compounds is another effective and low-cost approach to block the pores and other defects in the anodic films. Chemical conversion treatments by immersion in aqueous solutions containing salts such as Na_3_PO_4_ [264], Ca(NO_3_)_2_ [279], (Ce(NO_3_)_3_ [280,281], La(NO_3_)_3_ [278], Na_2_SnO_3_ [282], NaNd(SO_4_)_2_ [283], or Na_2_MoO_4_ [284] rely on the redox reactions that take place on the surface, producing compounds such as Ca_10_(PO_4_)_6_(OH)_2_ [279], La(OH)_3_ [278], CeO_2_/Ce_2_O_3_ [280], MgSnO_3_ [282], Nd_2_O_3_ [283], and MoO_3_ [284]. An advantage of the process is that high temperature curing is not needed after sealing.

Another successful approach is the incorporation of corrosion inhibitors into the PEO pores by sample immersion in the inhibitor solution under low-pressure conditions. It has been proven that PEO pores, as a high capacity reservoir for corrosion inhibitors, can provide long-term, active protection for Mg substrates [136]. For a deeper analysis of the corrosion inhibition mechanism using organic inhibitors added as a post-treatment, the reader is referred to Part III of this review [3]. The application of stable compounds such as SiO_2_ [86], TiO_2_ [285], and SiO_2_-ZrO_2_ [286] via sol-gel has also proved to be successful. The idea is to form a top barrier layer by covering the PEO surface and filling the micropores. Sol-gel coatings are not incorporated into the inner parts of the coating and rarely interact with the PEO coating material in an active way. Morphologically, sol-gel coatings are more uniform than conversion coatings produced by simple immersion, although cracking is likely to occur on their surface when thick multi-layers are produced. A typical practice in sol-gel treatments is to repeat the immersion/heat-treatment/drying sequence several times in order to obtain a coating that is thick enough to sufficiently cover the pores of the PEO layer [86,285,286,287].

Another novel approach consists of hydrothermal post-treatment, resulting in the formation of layered double hydroxides (LDH) [288]. LDHs, with a chemical formula [M^2+^_1−x_M^3+^_x_(OH)_2_][A^n−^]_x/n_·zH_2_O, are composed of positively charged brucite-like layers, containing both Mg^2+^ and Al^3+^, separated by regions containing anions and solvation molecules. LDH flakes can easily seal the pores of PEO coatings, but their greatest advantage is the possibility of loading their interlayer spaces with inhibitors which are released when triggered by chemical changes in the environment. This was first demonstrated on PEO-treated aluminum alloy, bringing about a remarkable increase in the corrosion resistance achieved through vanadate-intercalated LDH post-treatment [289].

The first directly grown LDH on PEO of Mg alloys without autoclave was achieved by Petrova et al. [290]. Currently, LDH post-treatment is being successfully exploited for the fabrication of composite PEO/inhibitor-loaded LDH coatings on Mg alloys in an increasing number of works [291,292,293,294,295,296,297]. Chen et al. investigated PEO/LDH systems with a variety of cations in the LDH layer, such as Ni-LDH, Zn-LDH, Al-LDH, and MgFe-LDH [294,295], of which Ni-LDH was found to form a continuous LDH layer over the PEO surface and produce an order of magnitude increase in total impedance (up to 5 × 10^5^ Ωcm^2^) compared with a stand-alone PEO coating. It was also shown that PEO coatings modified with composite Zn-Al LDH with reduced graphene oxide (rGO) nanosheets could effectively enhance corrosion resistance and reduce *i_Corr_* to 5 nA/cm^2^ [298]. Alternatively, in contrast to the direct synthesis method, LDH nanocontainers can be in situ incorporated into PEO coatings [299] or can be formed as a secondary reaction product of the primary deposited MnOOH film, spontaneously reacting with the corrosion-produced Mg^2+^ and OH^−^ [300]. Apart from the sealing effect of the LDH layer that improves the barrier properties of the PEO coating, the abilities to exchange the anionic inhibitors between its hydroxide layers and release them “on demand” offer active corrosion protection. More details on the active inhibition properties of LDH are provided in Part III of this review [3].

Superhydrophobic composite coatings have been fabricated on magnesium alloys by combining micro-arc oxidation (MAO) with post-treatments such as cyclic assembly in phytic acid and Ce(NO_3_)_3_ solution [301], stearic acid ethanol solution [302], alkynol inhibitor loading followed by hydrophobic wax application [303], hydrothermal treatment in boiling water followed by self-assembled monolayer formation in perfluorodecyltrichlorosilane (FDTS) [304], LDH treatment followed by perfluorodecyltriethoxysilane (PFDS) and perfluoropolyether (PFPE) lubricant infusion to form so-called slippery liquid-infused porous surface (SLIPS) [305], with some of these approaches maintaining remarkably high corrosion resistance for up to 21 days (Table 6). The application of fluorocarbons, such as polytetrafluoroethylene and fluoroparaffines [306,307,308,309], produced changes in impedance modulus and corrosion current density of up to two orders of magnitude (increase and decrease, respectively) and contact angles of 130°–152°.

**Figure 18:**
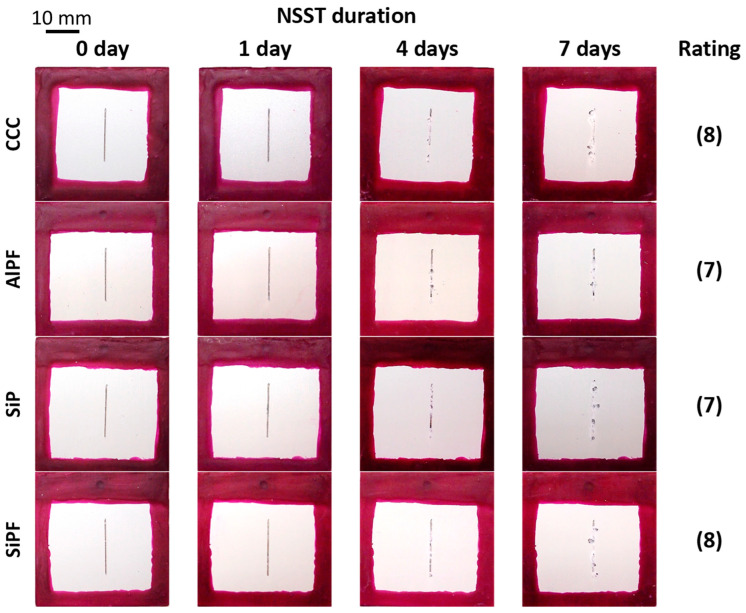
Salt spray testing results of Cr(VI)-based commercial coating and varied PEO covered with three-component epoxy primer, evaluated according to ASTM D 1654 standard [135].

**Figure 19:**
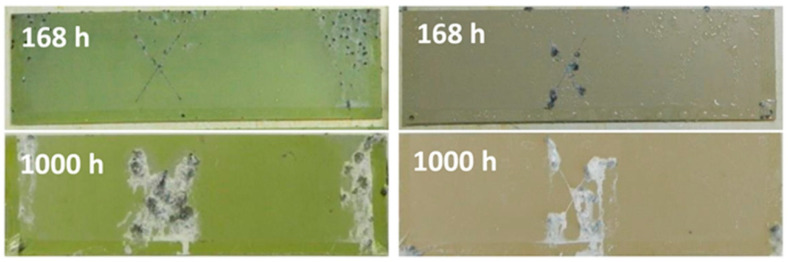
NSST results for full systems protection with scribed defect (in the form of a cross in the center): (left side) CCC/chromated primer system; (right side) inhibitor loaded PEO coating with chromate-free primer (SiPF/4MSA/primer) [136].

The application of polymeric compounds, which infiltrate the coating pores, is a common practice for anodized Mg components [310]. Research on this topic has gone in several directions, including silanes [274,311] and E-coatings [312,313,314]. For instance, silane treatment KH550 has been shown to enhance the corrosion resistance of a magnesium alloy with a silicate-based PEO coating [315]. E-coatings are widely applied in the automotive industry; they consist of the deposition of epoxy, epoxy polyester, or polyester resins by electrostatic powder spraying. For instance, AM60B alloy anodized at 70 V for 15 min in a strongly alkaline bath (KOH, Na_3_PO_4_, KF, Al(NO_3_)_3_), subjected to electrostatic powder spraying and curing (190 °C, 20 min), displayed no evidence of corrosion after salt spray testing for up to 595 h for a polyester paint [316]. Wierzbicka et al. reported that varied FPEO coatings covered with a three-component epoxy primer after a week of NSST were rated at up to 7 or 8 (out of a maximum of 10, according to the ASTM D 1654 standard) (Figure 18). These results are comparable with the rating of Cr(VI)-based commercial conversion coatings used in the aircraft industry [135]. The same authors demonstrated that FPEO coating loaded with 4-MSA inhibitor and coated with epoxy-based chromate-free primer with an active inhibition system based on lithium leaching technology outperformed a commercial Cr(VI)-based conversion coating with chromate-based primer after 1000 h of exposure in NSST (Figure 19) [136].

E-coating baths have also been shown to be suitable for the sealing of PEO coatings by a simple immersion process in an E-coating bath consisting of an epoxy resin and titanium dioxide [317].

**Figure 20:**
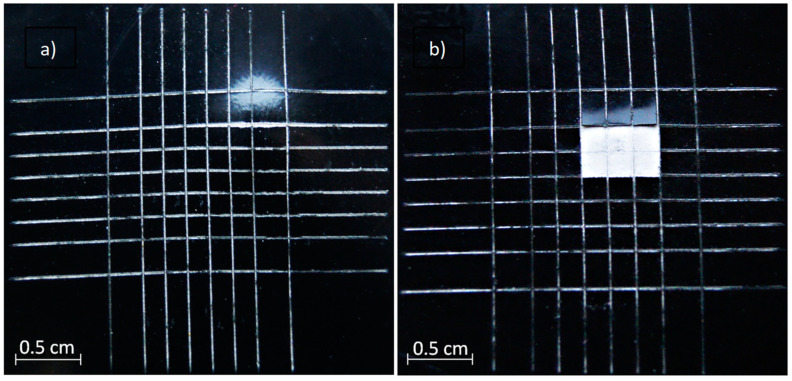
Surface appearance of (**a**) Ti/Zr + polymer and (**b**) PEO + polymer coatings after impact + adhesion tests [63]. Reprinted from [63] with permission from Elsevier.

In [63], the mechanical and corrosion performance of a polymer-coated AZ31 magnesium alloy pre-treated by plasma electrolytic oxidation (PEO) was compared with that of a polymer coated fluorotitanate–zirconate conversion coating (Gardobond X4707) on the same alloy. The electrostatically sprayed polymer was composed of polyester resin (50 wt.%), triglycidyl isocyanurate, polypropylene wax (≤1 wt.%), inorganic pigments, and fillers (barium carbonate and barium sulfate). Tests showed that both coatings (PEO and conversion coating) passed the adhesion test (rating 0); however, the results of impact and impact + adhesion tests revealed better performance of the coating with Ti/Zr treatment (Figure 20).

**Figure 21:**
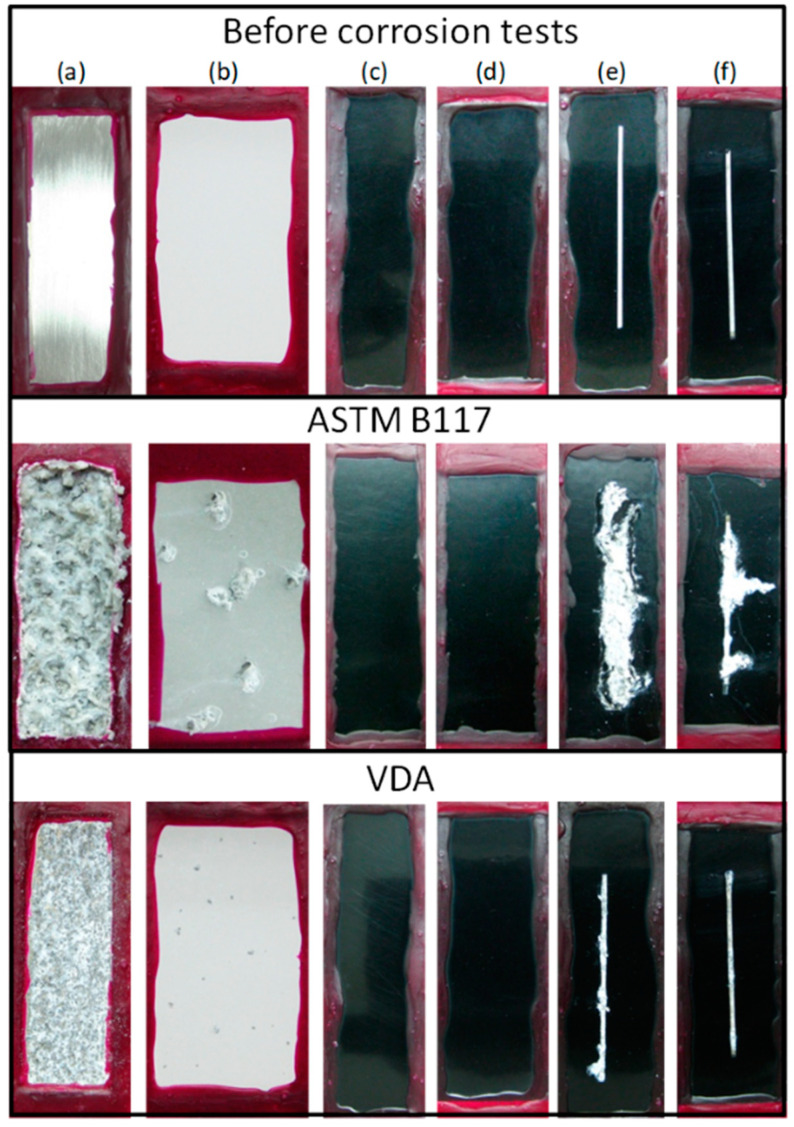
The surface appearance of AZ31 specimens before and after accelerated corrosion tests: (**a**) untreated, (**b**) PEO, (**c**) Ti/Zr + polymer, (**d**) PEO + polymer, (**e**) scribed Ti/Zr + polymer and (**f**) scribed PEO + polymer [63]. Reprinted from [63] with permission from Elsevier.

**Figure 22:**
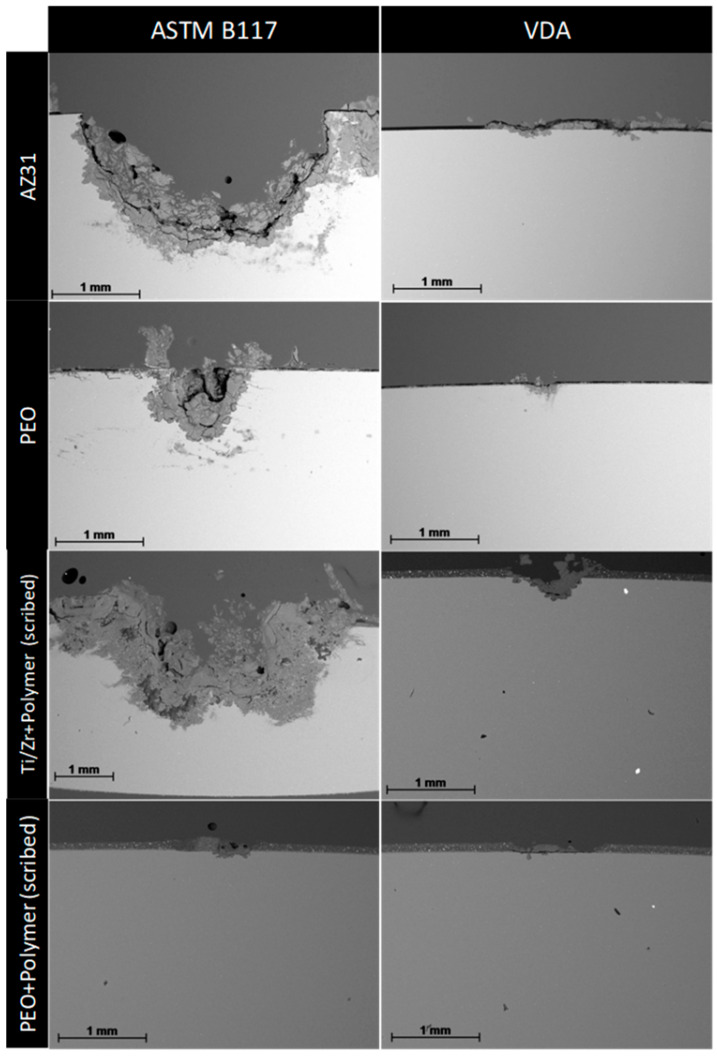
BSE cross-sections of AZ31 specimens after exposure to ASTM B117 and VDA tests [63]. Reprinted from [63] with permission from Elsevier.

Atmospheric corrosion tests, as per ASTM B117, and cyclic exposure to salt fog, as per VDA 621-415, revealed no damage either in the PEO or Ti/Zr treatments (Figure 21c,d). Greater differences were observed on scribed (ASTM D1654, procedure A) specimens, with the PEO + polymer coating showing a higher rating number, i.e., superior performance, than the Ti/Zr + polymer coating (Figure 21e,f). The superior anticorrosion properties of the PEO coating can be easily observed in Figure 22, which presents cross-sectional views of the studied samples after scribing and corrosion tests.

Other examples of polymer top coats enhancing the corrosion resistance of PEO coatings are multiple immersion in a titanium-based organic polymer [318] and sealing with hybrid epoxysilane [319], low-molecular weight polymers (e.g., MALPB, maleic anhydride-*g*-liquid polybutadiene) [320], and epoxy resins [321], which can be filled with inhibitor loaded nano- or micro-carriers, such as Ce^3+^ loaded zeolite microparticles [314] to ensure self-healing.

Post-treatment procedures and their effect on the PEO coating corrosion performance are presented in Table 6.

**Table 6:** PEO process parameters with post-treatment procedures and their effect on corrosion performance.

Post-Treatment/Introduced Species	Alloy Electrolyte PEO Treatment Conditions	Post-Treatment	Thickness/ Phases	Corrosion Data	Ref.
Ce(NO_3_)_3_ (CeO_2_, Ce_2_O_3_)	AM50 Na_2_SiO_3_, KOH AC square waveform 420/−60 V, 500 Hz, 10 min, 10 °C	Immersion Ce(NO_3_)_3_ 3 g/L, 20 min, 10 g/L, 20 min 10 g/L, 3 h H_2_O_2_, H_3_BO_3_, 30 °C	PEO 30–40 μm Post-treatment dissolution ~10–15 µm Mg_2_SiO_4_, MgO CeO_2_, Ce_2_O_3_	↑ Ce(NO_3_)_3_, ↑ t_sealing_→↑ R_corr_ (EIS)	[280]
La(NO_3_)_3_ (La(OH)_3_)	AZ91 Na_2_SiO_3_,NaOH, Na_5_P_3_O_10_ DC 0.5 A/cm^2^, 2 min	Immersion 12 g/L La(NO_3_)_3_ 30, 40, or 50 °C 10 or 30 min	PEO 30 µm MgO, Mg_3_(PO_4_)_2_, Mg_2_SiO_4_, La(OH)_3_	PDP in 0.1 M Na_2_SO_4_, 0.05 M NaCl (E_corr_; i_corr_) Untreated: −1.88 V; 2 × 10^−5^ A/cm^2^ PEO: −1.87 V; 1.5 × 10^−6^ La 30 °C 10 min: −1.73 V; 8 × 10^−7^ A/cm^2^ La 30 °C 30 min: −1.83 V; 4 × 10^−7^ A/cm^2^ La 40 °C 10 min: −1.82 V; 5 × 10^−7^ A/cm^2^ La 40 °C 30 min: −1.69 V; 2.8 × 10^−7^ A/cm^2^ La 50 °C 10 min: −1.73 V; 9 × 10^−7^ A/cm^2^ La 50 °C 30 min: −1.65 V; 2.8 × 10^−7^ A/cm^2^	[278]
Na_2_MoO_4_ (MoO_3_)	Mg-Li NaSiO_3_, NaOH, triethanolamine DC_pulsed_5 A/dm^2^, 2000 Hz; 15% duty cycle,10 min	Immersion 20 g/L Na_2_MoO_4_, 4 g/L NaF, 30 wt.% H_2_O_2_, 50 °C, 2 h	PEO and PEO-Mo 25 μm NaMgF_3_, Mg_2_SiO_4_, MgO, MoO_3_	PDP_5min_ in 3.5 wt.% NaCl (E_corr_; i_corr_) Untreated: −1.685 V; 7.045 × 10^−4^ A/cm^2^ PEO: −1.446 V; 6.4 × 10^−7^ A/cm^2^ PEO-Mo: −1.354 V; 2.5 × 10^−7^ A/cm^2^	[284]
Ce(NO_3_)_3_, Na_2_SnO_3_, Octadecylphosphonic acid (ODP)	AZ91D Na_2_SiO_3_, KOH, NaF AC 420 V/60 V, 500 Hz 200 mA cm^−2^, 200 s	Immersion Ce(NO_3_)_3_, 180 min 30 °C; Na_2_SnO_3_ 30 min 80 °C; 0.1672 g L^−1^ ODP in ethanol 24 h 23 °C	13 µm	Surface damage area after 7 days NSST (ASTM B117) PEO: 9.2 ± 2.4% PEO-Ce: 1.9 ± 1.3% PEO-Sn: 5.2 ± 0.5% PEO-ODP: 1.1 ± 0.1% EIS in 0.5 wt.% NaCl (|Z|_10mHz_) 3 h/7 days: PEO-Ce: 10^6/^2 × 10^5^ Ωcm^2^ PEO-ODP: 7 × 10^6/^3 × 10^4^ Ωcm^2^	[263]
Self-healing 8-hydroxyquinoline (8 HQ)	MA8 Na_2_SiO_3_, NaF bipolar pulses, anodic voltage 30 to 300 V, rate 0.45 V/s; cathodic pulse potentiostatic 30 V, 50% duty cycle, 300 Hz, 10 min, 25 °C	Immersion 3 g/L 8HQ (8-hydroxyquinoline C_9_H_7_NO) 120 min dried 140 °C, 20 min	16 µm	PDP_15 min_ in 3 wt.% NaCl (E_corr_; i_corr_) Untreated: − 1.59 V; 5.3 × 10^−5^ A/cm^2^ PEO: −1.51 V; 8.1 × 10^−7^ A/cm^2^ PEO-8HQ: − 1.44 V; 8.6 × 10^−8^ A/cm^2^ H_2_ evolution in 3 wt.% NaCl-40 days (P_corr_) Untreated: 1.133 mm/year PEO-coating: 0.154 mm/year PEO-8HQ: 0.128 mm/year	[322]
Self-healing NH_4_NO_3_ (LDH)	AZ31 Na_2_SiO_3_, KOH, KF DC_pulsed_0.3 A/cm^2^, 800 Hz, 10% duty cycle, V_end_360 V.	Immersion 0.02 M NH_4_NO_3_ (pH 12.8) 120 °C for 12 h	PEO 6.5 μm PEO-LDH 7 μm MgO, Mg(OH)_2_, LDH (Mg-Al LDH)	PDP_400 s_ in phosphate buffer saline-PBS 37 °C (E_corr_; i_corr_) Untreated: −1.45 V; 1.66 × 10^−5^ A/cm^2^ LDH: −1.12 V; 3.34 × 10^−5^ A/cm^2^ PEO: −1.22 V; 9.45 × 10^−6^ A/cm^2^ PEO-LDH: −1.2 V; 3.92 × 10^−6^ A/cm^2^	[288]
Inhibitors Ce(NO_3_)_3_ Ce^3+^ ions or 8- hydroxyquinoline (8HQ) Sol-gel TiO_2_ + (GPTMS). silane-based alkosol	ZK30 NaOH, Al(OH)_3_, Na_3_PO4, KF DC 125 mA/cm^2^,70 V, 10 min	Immersion 0.005 M Ce(NO_3_)_3_ or 0.005 M (8-hydroxyquinoline C_9_H_7_NO) 8HQ, 30 min Sol-gel dip-coating TiO_2_,(3-glycidoxypropyl)-trimethoxysilane (GPTMS) 1:2 vol., 100 s. cured 120 °C, 80 min	anodized film 0.7–3.0 µm sol-gel 3–4 µm	R_corr_: ZK_Anod_Ce^3+^_SG > ZK_Anod_SG > ZK_8HQ_SG > ZK_Anod anodized alloy = ZK_Anod sol-gel sealed = SG immersed in Ce^3+^ or 8HQ = Ce^3+^ or 8HQ	[323]
Inhibitor 1,2,4-triazole, Sol-gel TiO_2_ + silane-based sols (GPTMS)+ (PTMS)	ZE41 Na_2_SiO_3_, KF, NaOH poly(ethylene oxide), DC3 mA/cm^2^, 12 min, 20 ± 2 °C	Immersion 0.01 M 1,2,4-triazole, 15 s Sol-gel dip-coating TiO_2_, (3-glycidoxypropyl)-trimethoxysilane (GPTMS), phenyltrimethoxisilane (PTMS), 40 s suspended at room temperature, relative humidity 60% in open air, 1 h cured 120 °C,1.5 h	anodized film 1.8 ± 0.1µm sol-gel 6.3 ± 0.2 µm	R_corr_: ZE_Anod_Tr_SG > ZE_Anod_SG > ZE_SG substrate = ZE sol-gel sealed = SG anodizing = Anod immersed in 1,2,4-triazole = Tr	[324]
KH_2_PO_4_ (P) Na_2_SiO_3_ (Si) sol-gel SiO_2_	AM50B AM60B KOH, NaAlO_2_, K_3_PO_4_ DC 20–30 A/dm^2^ 7 or 14 min	Immersion 12% KH_2_PO_4_ (P) 60 °C, 5 min Immersion 5% Na_2_SiO_3_ (Si) 95 °C, 15 min Sol-gel sealing 14 mL tetra-ethylortho-silicate (TEOS), 2 wt.% Methyl-triethoxysilane (MTES), 1.2 mL ethanol, 2.5 mL H_2_O, 0.35 mL HCl, 1 min, annealing 160 °C, 3 h, ×3 repeated	10 or 25 µm MgO, MgAl_2_O_4_	PDP in 3.5 wt.% NaCl (E_corr_; i_corr_) AM50B Untreated: −1.55 V; 1.2 × 10^−5^ A/cm^2^ 10 µm PEO: −1.52 V; 1.45 × 10^−7^ A/cm^2^ 25 µm PEO: −1.47 V; 1.65 × 10^−7^ A/cm^2^ PEO-P: −1.62 V; 2.6 × 10^−8^ A/cm^2^ PEO-Si: −1.45 V; 2.4 × 10^−8^ A/cm^2^ PEO- SiO_2_: −1.39 V; 6.57 × 10^−9^ A/cm^2^ AM60B Untreated: −1.55 V; 8.2 × 10^−6^ A/cm^2^ 10 µm PEO: −1. 54 V; 1.04 × 10^−7^ A/cm^2^ 25 µm PEO: −1.56 V; 1.51 × 10^−7^ A/cm^2^ PEO-P: −1.67 V; 3.2 × 10^−8^ A/cm^2^ PEO-Si: −1.49 V; 2.2 × 10^−8^ A/cm^2^ PEO-SiO_2_: −1.44 V; 1.2 × 10^−8^ A/cm^2^	[86]
sol-gel TiO_2_	AZ91D NaAlO_2_, KOH DC 25 mA/cm^2^ 25 min, 25–30 °C	Sol-gel sealing tetra-n-butyl orthotitanate (TBT), ethanol, ethyl acetoacetate, 1 min, ×3 repeated heated 150 and 350 °C,1 h	PEO 4µm PEO-TiO_2_ 6µm MgO, MgAl_2_O_4_, MgTi_2_O_5_	PDP in 3.5 wt.% NaCl (E_corr_; i_corr_) Untreated: −1.509 V; 2.352 × 10^−6^ A/cm^2^ PEO: −1.479 V; 1.607 × 10^−6^ A/cm^2^ PEO-TiO_2_-(150 °C): −1.316 V; 7.96 × 10^−8^ A/cm^2^ PEO-TiO_2_-(350 °C): −1.261 V; 4.838 × 10^−7^ A/cm^2^	[285]
sol-gel SiO_2_-ZrO_2_	AZ91D NaAlO_2_, NaOH, small quantity of Montmorillonite and acacia gum pulsed voltage, increased to 180–200 V, then 0.5 h	Sol-gel sealing stoichiometric amounts of ethyl silicate, zirconyl chloride octahydrate ethanol, 1 min drying 150 °C, 1 h	Sol-gel layer 5 µm	PDP_10min_ in 3.5 wt.% NaCl (E_corr_; i_corr_) Untreated: −1.428 V; 3.395 × 10^−5^ A/cm^2^ PEO: −1.326 V; 3.921 × 10^−7^ A/cm^2^ PEO- SiO_2_-ZrO_2_: −0.406 V; 1.577 × 10^−9^ A/cm^2^	[286]
Inhibitor loaded and sol-gel sealed	AZ91 Na_3_PO_4_, KOH DC pulsed: t_on_:t_off_ = 1:9, 250 Hz, 40 mA cm^−2^, 60 s	Inhibitors: Na glycolate (Gly), Na 4-aminosalicylate (4AmSal), Na 2,6-pyridinedicarboxylate (PDC). Sol-gel: (3-glycidoxypropyl)-trimethoxysilane (GPTMS) and Ti (IV) propoxide (TPOT)	2.5 µm PEO + 2.5 µm sol-gel	EIS in 0.5 wt.% NaCl (|Z|_10mHz_), 2 h/336 h PEO-SG: 10^8^ Ωcm^2^/10^4^ Ωcm^2^ PEO-Gly-SG: 10^7^ Ωcm^2^/10^7^ Ωcm^2^ PEO-4AmSal-SG: 10^8^ Ωcm^2^/2 × 10^7^ Ωcm^2^ PEO-PDC-SG: 10^8^ Ωcm^2^/3 × 10^6^ Ωcm^2^	[325]
Inhibitor loaded halloysite nanotubes (HNT)	AZ31 Na_2_SiO_3_, KOH, NaF Pulsed DC: 5000 Hz, 10% duty cycles, 40 mA cm^−2^, 10 min	Immersion: 10 min in an aqueous solution 20 g L^–1^ of inhibitor loaded HNTs, 22 °C. Inhibitors: 8-hydroxyquinoline (8HQ), ammonium molybdate (Mo), ammonium metavanadate (V)	30 µm PEO MgO, MgSiO_4_	LEIS in 0.05 M NaCl up to 34.1 h over 300 × 2000 μm and 250 × 500 μm artificial defects All PEO-HNT-inhibitor coatings provided self-healing of small defects; only PEO-HNT-V partially restored large defect.	[252]
Self-healing PEO-Ce-LDH-P	AZ31 NaAlO_2_, NaOH, AC: 100 Hz, +250 V/−50 V, 26% duty cycle, 600 s	Ce-conversion coating: 50 °C, 2 h; LDH: NaNO_3_ 125 °C, 12 h; Phytic acid immersion: pH11, 80 °C, 1 h	PEO-Ce 1.2 µm PEO-Ce-LDH 2.7 µm PEO-Ce-LDH-P 2.8 µm	EIS in 3.5 wt.% NaCl (|Z|_10mHz_) PEO-Ce-LDH-P 30 min: 10^5^ Ωcm^2^ 7 days: 2 × 10^5^ Ωcm^2^ 21 days: 10^6^ Ωcm^2^	[292]
Superhydrophobic PEO-HDT-FTDS-oil impregnation	AZ31B NaSiO_3_, KOH, KF DC: 100 mA cm^−2^ 10 min	HDT: 100 °C H_2_O 50 min + Hydrophobization with FDTS and oil impregnation by solvent exchange with Krytox GPL 103	~20 µm Wettability PEO-HDT-FTDS: 173° PEO-HDT-FTDS-oil: 123°	EIS in 3.5 wt.% NaCl (|Z|_10mHz_), 0/15 days PEO-HDT-FTDS: 5 × 10^6^ Ωcm^2^/2 × 10^6^ Ωcm^2^ PEO-HDT-FTDS-oil: const. ~10^8^ Ωcm^2^	[304]
Superhydrophobic PEO-LDH-SLIPS	AZ91D NaSiO_3_, NaOH Pulsed DC 30 mA cm^−2^, 100 Hz, duty cycle 10%, 300 s.	+Mo-intercalated Mg-Al-LDH, 120 °C, 8–48 h + PFDS + PFPE	~9 µm 121° wettability	EIS in 3.5 wt.% NaCl (|Z|_10mHz_) 0 days: 2 × 10^8^ Ωcm^2^ 18 days: 2 × 10^6^ Ωcm^2^ Water repellance, active protection by Cl^-^ and MoO_4_^2-^ exchange, regeneration of barrier layer	[305]
Silane coupling agent (SCA)	99.9% Mg silicate electrolyte 300 V, 500 Hz, 2.5% duty cycle, 10 min, ultrasonic frequency 60 kHz	Immersion NaOH (1, 2, 3 mol/L), 60 °C, 1 h Immersion silane coupling agent (SCA) KH550 C_2_H_5_OH, H_2_O, 1:9:1 vol. heated	-	PDP in 0.9 wt.% NaCl (E_corr_; i_corr_) PEO: −1.517 V; 2.135 × 10^−2^ A/cm^2^ PEO-NaOH(1M)-SCA: −1.488 V; 2.73 × 10^−3^ A/cm^2^ PEO-NaOH(2M)-SCA: −1.464 V; 8.927 × 10^−4^ A/cm^2^ PEO-NaOH(3M)-SCA: −1.442 V; 2.383 × 10^−4^ A/cm^2^	[315]
E-coating electroless	ZE41 Tagnite treatment up to ~20 μm thick film	E-coating bath solution (water 71–82 wt.%, epoxy resin 16–26 wt.%, TiO_2_ 1.3 wt.%), 10 s, baked 171 °C, 25 min	PEO 20 μm	PDP in 5 wt.% NaCl (E_corr_; i_corr_) PEO: 10^−2^ –10^−3^ mA/cm^2^ Sealed:10^−6^ –10^−6^.^5^ mA/cm^2^ ↑E_corr_	[317]
Titanium organic polymer (TOP)	AZ91D Na_2_SiO_4_, KOH DC_pulsed_, 360–400 V, 1–2 h	Immersion titanium organic polymer (TOP), solvent 1:20, applied vacuum, 1 min, cured 50 °C, 30 min ×2, 3 repeated	PEO 20 µm sealing layer 3–5 µm	PDP in 3.5 wt.% NaCl (E_corr_; i_corr_) Untreated: −1.574 V; 3.176 × 10^−5^ A/cm^2^ PEO: −1.555 V; 2.962 × 10^−7^ A/cm^2^ PEO-TOP: −1.119 V; 4.107 × 10^−10^ A/cm^2^	[318]
Epoxy-silane (ES)	ZE41 NaOH, Na_2_SiO_3_, KF 7.5 g/L poly(ethylene oxide) DC 3 mA/cm^2^, 9 min, 20 °C	Immersion silane (0.14 M), epoxy (0.98 M), DETA amine (0.37 M), ethanol, and acetone 5 s ×4 repeated cured 150 °C, 1.5 h	PEO 1.8 µm PEO-ES 10 µm	PDP_10 min_ in 0.5 M NaCl (E_corr_; i_corr_) Untreated vs. PEO: ↓i_corr_ by 2–4 orders of magnitude Immersion in 0.6 M NaCl for 31 days Untreated: filiform corrosion, multiple pits, most commonly located at the edge. PEO-ES: no signs of corrosion onset nor any indication of coating delamination	[319]
Polymer (MALPB)	AZ31B KOH, Na_3_PO_4_, KF, Al(NO_3_)_3_ DC 5 mA/cm^2^ up to 80 V 30 min, 30 °C	Immersion Maleic anhydride-g-poly(1,2-butadiene) polymer (MALPB) Mn = 1020 1 min cured 30 to 180 °C (10 °C/min) 15 min	PEO 3.5 µm, MALPB 10 µm	PDP_30 min_ in 3.5 wt.% NaCl (E_corr_; i_corr_) MALPB: −1.35 V PEO: −1.48 V PEO-MALPB: −1.19 V NSST-scratch test 50 g/L NaCl, 1–2 mL/h, 200, 400 h corrosion PEO-MALPB < MALPB PEO-MALPB almost unchanged after 200 h, smaller spoiled dots after 400 h	[320]
Epoxy resin Ni-P	AZ31 Na_2_SiO_4_, KOH 600 V, 600 Hz, 10% duty cycle, 20 °C, 10 min	Immersion commercial epoxy resin (24 h drying) Pretreatment: -NaOH, 75 °C,40 min -silane solution, 20 °C, 3 min, -cured 100 °C, 30 min. -PdCl_2_, HCl, 20 °C, 1 min -NaH_2_PO_2_, 20 °C, 10 min Electroless Ni–P plating (25 g/L NiSO_4 ×_ 6H_2_O, 23 g/L NaH_2_PO_2_·H_2_O, 20 g/L, Na_3_C_6_H_5_O_7_·2H_2_O, 24 g/L NH_4_F, 3 mg/L CS(NH_2_)_2_, 75 °C 20 min	Ni-P 5 μm polymer 130 μm PEO 8 μm	PDPin 3.5 wt.% NaCl (E_corr_; i_corr_) PEO: −1.444 V; 8.826 × 10^−8^ A/cm^2^ PEO-epoxy: −1.357 8.594 × 10^−9^ A/cm^2^ PEO-epoxy-Ni-P: −0.416 V; 5.979 × 10^−6^ A/cm^2^ EIS immersion time, pitting onset: PEO-epoxy 408 h PEO-epoxy-Ni-P 624 h	[321]

Based on the corrosion data, post-treatments are the most effective way to improve the corrosion resistance of anodic films on Mg alloys. Corrosion current density values in the order of 10^−9^–10^−10^ A cm^−2^ can be achieved [286,318]. The top coat not only seals the pores but also forms an additional layer that isolates the substrate from external aggressive factors. The thickness of top coats can be up to 130 µm [321]. Methods providing a self-healing effect, such as inhibitor loaded LDH nanocontainers, deserve special attention and should be explored in more detail, since they can actively protect Mg substrates during service by repairing small defects. Some disadvantages of post-treatments are the longer time required to obtain the final product and added cost of the high temperature drying/curing steps which are common in these processes.

## 6. Perspectives

In summarizing the recent advances, it is evident that the combined use of protective strategies, i.e., the enrichment of porous PEO layers with corrosion inhibitors, followed by sealing with hybrid or organic coatings loaded with an inhibitor, achieves enhanced long-term corrosion protection for magnesium alloys, providing a strong barrier (when the coating in intact) and active corrosion protection in case the coating is damaged. The protection level of such a system is comparable to that of the gold standard for many decades—a chromate-based conversion layer coated with an organic chromate-based primer. The fault tolerance of the composite coating is the result of the cumulative performance of all its components, i.e.,: i) the anodic film, which provides barrier protection and serves as the repository for ii) corrosion inhibitors that ensure active corrosion suppression; and iii) an organic coating that enhances the protective properties of the barrier, seals the porous anodic layer with inhibitors, and enhances the active corrosion inhibition due to its ability to inhibit the action of corrosive agents [136,326].

Further development can be expected of each element of such combined protection systems, which may also focus on coating multifunctionalization, such as abrasion, wear, thermal resistance, biocidal effect, superhydrophobicity, self-cleaning, etc. For anodic layers in particular, there is a large scope of future work to further minimize energy consumption. In these regards, organic electrolytes, as well as more concentrated alkaline electrolytes, should be explored to bring the coating formation voltage to below 150–200 V and limit the treatment times to under 60 s. Appropriate recycling strategies will have to be considered for such electrolytes in order to achieve sustainability and successful integration into the circular economy. For sealing strategies, trends such as PEO/LDH and PEO/sol-gel combinations are showing great potential. LDH scaffolds can be easily loaded with smart inhibitors, but two major challenges of this strategy remain: (i) optimization of the PEO layer thickness and composition to facilitate the growth of LDH; and (ii) the search for suitable, environmentally friendly inhibitors that can be loaded into LDH. Sol-gel layers, on the other hand, can be loaded with smart nanocontainers, such as clay particles, multi-walled CNT, and mesoporous silica [327]. With regard to the latter in particular, there is a wealth of knowledge in the field of smart drug release for biomedical applications [9,328], but this appears to be almost completely overlooked when it comes to corrosion-resistant engineering applications. The adaptation of mesoporous silica to smart inhibitor release and making it part of a hybrid coating system, e.g., within the LDH layer [329] or organic top-coat [330], seem to be the next logical next steps. The paintability of such hybrid coatings is largely unknown and may present challenges of its own, for instance, due to the hydrophobic nature of some of the loaded inhibitors.

Last but not least, the rapid development of additive manufacturing technologies for magnesium alloys [331,332,333] (which, until recently, has been lagging behind those for other light alloy systems, such as aluminum and titanium, although this is a topic for a separate discussion) will inevitably spur research in the field of corrosion protection by anodic coatings, among other approaches. Such research is still in an embryonic state and so far has been limited exclusively to biomedical applications [334,335].

## Figures and Tables

**Table 1:** Summary of the conditions and corrosion properties of commercial anodic coating treatments and selected research results.

Name of Process	Electrolyte	Procedure Parameters	Composition of Coating	Thickness of Coating	Salt Spray Test	Ref.
Dow-17	NH_4_HF_2_, Na_2_Cr_2_O_7_, H_3_PO_4_	pH~5 70–80 °C AC 0.5–5 A/dm^2^ Thin coating: V_end_ 60–75 V, 4–5 min Thick coating: V_end_ 90–100 V, 25 min	MgF_2_, NaMgF_3_, Mg*_x_*_+*y*/2_O*_x_*(OH)*_y_* small amounts of Cr_2_O_3_	2.5–7.5 µm (thin coatings) 23–38 µm (thick coatings)	AZ91D Rating 5–14 days ZE41A Rating 0–2 days (ASTM D1654-Method B)	[22,60,69]
HAE	KOH, Al(OH)_3_, K_2_F_2_, Na_3_PO_4_, K_2_MnO_4_	pH ~14 27 °C AC 1.5–2.5 A/dm^2^ Thin coating: V_end_ 65–70 V, 7–10 min Thick coating: V_end_ 80–90 V, 60–90 min	-	5–10 µm (thin coatings) 25–80 µm (thick coatings)	AZ91 HP 25 µm (3–41 corrosion points/dm^2^ after 24 and 100 h) 40 µm (0–18 corrosion points/dm^2^ after 24 and 100 h)	[22,60,77]
Tagnite	Alkali hydroxide, metal fluorides, alkali metal fluorosilicates, hydrogen fluorides,	10–20 °C DC_pulsed_ 1–5 A/dm^2^ V_end_ 200–400 V	MgO with minor surface deposition of hard fused silicates	5–10 µm (thin coatings) 20–25 µm (thick coatings)	ZE41 Rating 9: 24–200 h (ASTM D1654-Method B) Far superior compared to HAE and Dow 17	[22,27,60]
Anomag	NH_3_, NaNH_4_HPO_4_	RT DC 10 mA/cm^2^ 170–350 V	MgO–Mg(OH)_2_, some additives like Mg_3_(PO_4_)_2_depending on bath composition	3–8 µm 10–15 µm 20–25 µm	AZ91 3–8 µm Rating 9: 150 h 10–15 µm Rating 9: 400 h 20–25 µm Rating 9: 1300 h	[60,80,81]
Keronite	different alkaline solutions	20–50 °C Bipolar signal	Mostly MgAl_2_O_4_, minor content of SiO_2_ and SiP_2_	35 µm	AZ91D Die cast Rating 9: 1000 h with a polymer top-coat (ASTM D1654-92 Method B)	[60,82]
MagOxid	-	RT DC 1–2 A/dm^2^ 400 V Thick coating: V_end_ 90–100 V, 25 min	MgO, Mg(OH)_2_, MgF_2_ and MgAl_2_O_4_	5 µm (thin coatings) 30 µm (thick coatings	AZ91HP 80–100 h (DIN EN ISO 10 289)	[28,81]
SweetMag	alkaline solution, free from chromates, borates and fluorides	25 °C DC 2 A/dm^2^ V_end_ < 180 V (aversge); < 300 V (max) 18 min	-	20 µm	-	[83]
	KOH, Al_2_(OH)_3_, KF, Na_3_PO_4_ varied additives: chromate, tungstate, vanadate, stannate, manganate (HAE bath)	DC 15 A ft^−2^, 90 min, 24 °C optional post-treatment 45 s immersion NH_4_HF_4_ + Na_2_Cr_2_O_7_ aging procedure 4 h at 100% relative humidity 175–180 °C	-	-	FS1 (dichromate pickle) ASTM B-117-44T 48, 120, 312 h corrosion: vanadate <stannate <chromate <no-additive <tungstate <manganate ↓corrosion after post-treatment (all electrolytes) vanadate < no-additive < manganate < chromate < tungstate < stannate	[84]
	KOH, Na_2_CO_3_, Na_2_SiO_3_, Na_2_B_4_O_7_	5–85 °C, current density 5–500 mA/cm^2^, 150 V_end_, 10–80 min	-	10–53 µm	AZ91D 336 h 14 µm: Rating 7–8 30 µm Rating 9 53 µm: Rating 9 336 h (ASTM B893-98) Far superior compared to HAE (Rating 2–3) and Dow 17 (Rating 5–6 (15 µm) 8–9 (122 µm))	[85]

**Table 3:** Summary of energy input parameters during PEO of Mg alloys.

Variable	Process Parameter	Alloy Electrolyte	Effects	Corrosion Data	Ref.
100, 120, 140, 160 V 500, 1000, 1500, 2000 Hz 0.1, 0.4, 0.6, 0.9 duty cycle	AC square 30 °C, 3 min	AZ91D NaOH, H_3_BO_3_, Na_2_B4O_7_, C_6_H_5_Na_3_O_7_, organic additive	14.88–37.32 µm ↑voltage, ↑duty cycle → ↑thickness, ↑pores, and cracks ↑frequency → ↓thickness <120 V → Thin and transparent coating	140 V, 2000 Hz, 0.4 duty cycle (22.3 µm) → best EIS performance R_coat_ = 1.54 × 10^5^ Ωcm^2^	[139]
20,30,40 mA/cm^2^ 400,440, 480 V_end_ 200, 400, 600 Hz 15, 25, 35% duty cycle	20 °C, 700 s	AZ91HP HF, H_3_PO_4_,H_3_BO_3_, NH_3_	9–22 µm Factors influence on thickness: final voltage > current density > duty cycle > frequency. ↑final voltage → ↑porosity ↑frequency → ↓porosity	Effect of factors on R_corr_: voltage > frequency> duty cycle > current density 20 mA/cm^2^, 440 V, 600 Hz, 15 or 35% duty cycle → best (336 h of salt spray test (ASTM B117-95 and ASTM B537-70 with no evidence of pitting corrosion)	[140]
Voltage mode Two-step: 280 V-6 min + 360 V-9 min Three-step: 280 V-6 min +320 V-4,5 min + 360 V-4,5 min	DC_bipolar_(−20 V), 20% duty cycle, 600 Hz, 35 °C, t_total_ = 15 min	ZK60 NaAlO_2_, Na_3_PO_4_, NaOH, NaB_4_O_7_, C_6_H_5_Na_3_O_7_	Two-step 20.2 µm (denser) Three-step 15.76 µm (smoother)	Two-step → best R_corr_ Z_10mHz_ 1.414 × 10^5^ Ω cm^2^-order of magnitude higher compared with the three-step voltage mode	[141]
200, 400, 800, 1000, 1500 Hz	constant current density 5 A/dm^2^, 30 °C, 10 min	AZ91D Na_2_SiO_3_, NaF, NaOH	↑frequency → denser and thinner coatings (100 Hz–44.98 μm, 200 Hz–28.20 μm, 400 Hz–20.3 μm) ↑frequency →↓friction coefficient Wear resistance of the coatings is influenced by both the thickness and structures	↑frequency → ↑R_corr_	[142]
10, 100,1000 Hz	DC_pulsed_ 30 mA cm^−2^ t_on_/t_off_ = 1:9 10 °C, 30 min	AM50 KaOH, Na_3_PO_4_,	~73 µm for 10 Hz, ~45 µm for 1000 Hz ↓frequency → ↑growth rate, additional phases ↑frequency (1000 Hz) → ↑V_end_ (551 V-1000 Hz), smooth surface with fine microstructure/surface morphological features	10 Hz → best EIS performance Z_10 mHz-0.5 h_ 2.5 × 10^5^ Ω cm^2^ Z_10 mHz-50 h_ 1.1 × 10^5^ Ω cm^2^	[143]
Unipolar pulse(S1): I^+^ = 1A_400 µs on-100 µs off_ Bipolar pulse (S2): I^+^ = 1A_400 µs on-100 µs off_ I^−^ = 0.9A_400 µs on-100 µs off_ Bipolar pulse (S3): I^+^ = 1A_400 µs on-200 µs off_ I^−^ = 0.7A_600 µs on-100 µs off_ Bipolar pulse (S4): I^+^ = 1A_400 µs on-100 µs off_ I^−^ = 0.5A_600 µs on-100 µs off_	constant current control 25 °C, 45 min	AJ62 Na_2_Al_2_O_4_, KOH	S1 = 40–60 μm, S2 = 35–45 μm, S3 = 40–55 μm, S4 = 55–80 μm Unipolar → ↑porosity and microcracks Bipolar → thicker and denser inner layer and thin and porous outer layer. Cathodic pulses → helps to eliminate, or at least reduces, the strong discharges, reducing high temperature spikes. Temperature is still sufficient to form MgAl_2_O_4_	Bipolar (S2) → best EIS performance Z_100 mHz-0.5 h_ 2.8 × 10^6^ Ω cm^2^	[144]
DC, Unipolar pulse, Bipolar pulse	Positive pulse 250–400 V, negative pulse −20 V, 500 Hz, 15% duty cycle, 40 °C, 15 min	ZK60 Na_2_SiO_3_·9H_2_O, NaOH, NaF	Bipolar mode (400 V/−20 V) → shorter and smaller discharges → thicker and denser coating layer.	Bipolar 400 V/−20 V) → best I_corr_ in 3.5% NaCl, 6.7 × 10^−7^ A cm^−2^	[145]
Unipolar vs. Bipolar i^+^ = 30 mA/cm^2^ i^−^ = 10–20 mA/cm^2^	3000 Hz 10% duty cycle, 10 min	cp-Mg Ca(OH)_2_, Na_3_PO_4_	~25µm (unipolar), ~16 µm (bipolar 10 mA/cm^2^), unstable (bipolar 20 mA/cm^2^) 500 V_end_ (unipolar), 460 V_end_ (bipolar 10 mA/cm^2^) Bipolar → finer porosity and denser inner region, albeit the coating thickness reduced with increasing negative current density, but apparent macroscopic defects No qualitative difference in the chemical and phase compositions after the unipolar and bipolar pulses application	Unipolar Z_10 mHz-2 h_ 508 Ω cm^2^ Bipolar Z_10 mHz-2 h_ 113 Ω cm^2^	[146]
AC 50 Hz Positive pulses (PP) added during each positive AC interval Negative pulses (NP) added during each negative AC interval	5–12 A/dm^2^ t_pulses_ 0–8 ms, 20–25 °C, 60 min	MA2-1 NaOH, Na_5_P_3_O_10_	50–75 µm Positive and negative pulses → ↑efficiency of coating deposition Optimal combination appeared to be 6-4 ms for positive pulses and 2–6 ms for negative ones.	Droplet test in HCl + CuCl_2_ PP mode → longest time until potential change	[147]
Time, 1.5–10 min	DC_pulsed_ 30–50 mA/cm^2^ 380 V	AZ80 Na_2_SiO_3_, KOH, KF	No duty cycle data provided. 4–5 μm- thick coating in 3 min, dense inner layer, fine surface porosity.	Non-sealed 3 min coating–no corrosion in 80 h NSST, pitting at 100 h.	[145]

**Table 4:** Organic and inorganic electrolyte additives.

Additive		Alloy/ Electrolyte/ Process Parameter	Effects	Corrosion Data	Ref.
Inorganic	chromate, tungstate, vanadate, stannate, manganate (0.086 M each)	FS1 (dichromate pickled in manufactory) HAE bath (KOH, Al_2_(OH)_3_, KF, Na_3_PO_4_) DC 15 A ft^−2^, 90 min, 24 °C Optional post-treatment (1) 45 s immersion NH_4_HF_4_ + Na_2_Cr_2_O_7_ (2) 4 h at 100% relative humidity 175–180 °C	80–90 V_end_ varied colors similar hardness	ASTM B117-44T312 h corrosion: vanadate < stannate < chromate < no-additive < tungstate < manganate ↓corrosion after post-treatment (all electrolytes) vanadate < no-additive < manganate < chromate < tungstate < stannate	[84]
	KMnO_4_ 0.07 M	AZ91 KOH, KF, Na_2_SiO_3_ DC 100 mA cm^−2^, 1–5 min	↓thickness 11.19 to 4.84 µm MgO, MgF_2_, Mg_2_SiO_4_, Mn_2_O_3_ ↓pore population ↑conductivity 29.23 to 30.23 mS cm^−1^ ↓V_end_ 352 to 286 V	PDP in 3.5 wt.% NaCl (E_corr_; i_corr_) Untreated: 1.56 V; 1.33 × 10^−5^ A/cm^2^ 0 M, 5 min: −1.54 V; 1.78 × 10^−7^ A/cm^2^ 0.07 M, 5 min: −1.34 V; 6.95 × 10^−9^ A/cm^2^ ASTMB117 200 h 0 M severe pits0.07 M localized pits	[175]
	Na_2_MoO_4_ 0.3, 0.6, 1, 3 g/L	AZ91 Na_2_SiO_3_, NaOH, diethylamine DC 0.05 A cm^−2^, 15 min	1 µm (0.3 g/L), 40 µm (3 g/L) ↑V_end_ < 250 V MgO, Mg_2_SiO_4_, MoO_3_, MgMoO_4_ ↑uniform covering, (best 0.3 g/L Na_2_MoO_4_) ↑corrosion resistance with ↓molybdate content (but without molybdate is the worst)	PDP in 0.1 M Na_2_SO_4_ + 0.05 M NaCl (E_corr_; i_corr_) Untreated: −1.55 V; 1.5 × 10^−5^ A cm^−2^ 0 g/L: −1.39 V; 3.5 × 10^−5^ A cm^−2^ 0.3 g/L: −1.37 V; 1 × 10^−6^ A cm^−2^ 3 g/L: −1.41 V; 7 × 10^−6^ A cm^−2^	[176]
	K_2_SnO_3_ 0.1, 0.2, 0.5 M	AZ91D KOH, KF, Na_3_PO_4_ DC 10 mA/cm^2^, 10 min	↓thicknesses 7–10 μm to 5 μm ↑V_end_ < 65 V 0.8–2.9 at% Sn, MgSn(OH)_6_ ↑homogeneity, ↓porosity 0.5 M cracks appearance	PDP_24h_ in D1384-87 ASTM water 148 ppm Na_2_SO_4_; 138 ppm NaHCO_3_, 165 ppm NaCl (E_corr_) Untreated: −1.4 V 0–0.5 M: −1.55 to −1.6 V	[132]
	Na_2_WO_4_ 2, 4, 6, 8 g/L	AZ91HP NaOH, phytic acid Constant current density 40 mA/cm^2^, frequency 2000 Hz, duty cycle 20% 30 °C, 3 min	≈thickness (4.9 to 4.6–5.5 µm) ↓V_end_ 374 V (0 g/L) to 269 V (8 g/L) WO_3_/Na_2_WO_4_ ↑pore size ↑conductivity	Immersion in 3.5 wt.% NaCl for 24 h ↑Na_2_WO_4_→↓R_corr_ 0 g/L: one small corrosion pit 8 g/L: four corrosion pits, largest pit 8 mm × 6 mm	[189]
	CoSO_4_ 0.2, 0.4, 0.6, 0.8 g/L	AZ31B Na_2_SiO_3_, NaOH, NaF, KNaC_4_H_4_O_6_·4H_2_O, C_3_H_8_O_3_ DC pulsed: 100 Hz, duty cycle 20%, 5 A dm^−2^, 30 °C, 30 min	~8 µm MgO, Mg_2_SiO_4_, SiO_2_, ~1 at.% Co 0.4 g/L: increased hardness and thermal shock resistance (2000 cycles)	PDP in 3.5 wt.% NaCl (i_corr_) 0 g/L: 7.46 × 10^−9^ A/cm^2^ 0.4 g/L: 6.82 × 10^−9^ A/cm^2^ 0.6 g/L: 7.09 × 10^−9^ A/cm^2^ 0.8 g/L: 7.17 × 10^−9^ A/cm^2^	[190]
	Ce(NO_3_)_3_ 0.1 g/L La(NO_3_)_3_ 0.1 g/L	AZ31 Na_2_SiO_3_, NaOH AC asymmetrical pulse constant current density 5.0 A dm^−2^, 35 °C, 15 min	13.2 µm (0 g/L), 11.9 µm (Ce), 15.1 µm (La) V_end_ < 325 V (La > Ce > 0 g/L) MgO, MgSiO_3_ ≈ composition, morphology ↑compactness	PDP in 3.5 wt.% NaCl (E_corr_; i_corr_) Untreated: −1.45 V; 3.69 × 10^−5^ A/cm^2^ 0 g/L: −1,4 V; 1.34 × 10^−5^ A/cm^2^ Ce(NO_3_)_3_: −1.38 V; 1.26 × 10^−6^ A/cm^2^ La(NO_3_)_3_: −0.89 V; 7.84 × 10^−7^ A/cm^2^	[174]
	K_2_ZrF_6_ 5,10,15 g/L	AZ91D Na_2_SiO_3_, NaF, NH_4_H_2_PO_4_, C_6_H_5_O_7_Na_3_, AC 300 V, 480 Hz, duty cycle 30%, 45 °C, 10 min	5–8 μm MgO, MgF_2_, t-ZrO_2_, MgSiO_3_ and amorphous phosphate self-sealing effect ↑compactness ↓coating defects ↓porous outer layer thickness	PDP in 3.5 wt.% NaCl (E_corr_; i_corr_) 0 g/L: −1.503 V; 6.57 × 10^−6^ A/cm^2^ 5 g/L: −1.407 V; 9.77 × 10^−7^ A/cm^2^ 10 g/L: −1.483 V; 6.71 × 10^−7^ A/cm^2^ 15 g/L: −1.421 V; 4.04 × 10^−7^ A/cm^2^	[184]
	K_2_TiF_6_ 0, 2,4, 6, 8, 10, 12 g/L	AZ91D (NaPO_3_)_6_, NaOH, (HOCH_2_CH_2_)_3_N, positive pulse voltages, different current densities (RMS) 3–10 A/dm^2^, 200 Hz, duty cycle of 15%, 40 °C, 15 min	20 μm (0 g/L),32 μm (2.0 g/L), 39.8 μm (12.0 g/L) V_end_ = 545, 569, 545, 524 V, (0, 4, 8, 12 g/L) >6 g/L MgO, MgAl_2_O_4_, Mg_2_TiO_4_, Mg_2_PO_4_F, MgF_2_ >8 g/L TiO_2_- Anatase ↑uniformity (0–8.0 g/L) ↓pore diameter	ASTM B117, 240 h PEO 8 g/L, 6 A/dm^2^ A few corrosion spots were observed on the surface	[179]
	Mixture of K_2_ZrF_6_ 0.035 M Y(NO_3_)_3_ 0.002 M	AZ91D NaAlO_2_, KOH AC, anodic voltage 300 V, cathodic voltage −60 V, frequency 700 Hz, duty ratio 30% 30 °C, 5 min	~11 µm Al_2_O_3_, c-ZrO_2_, t-ZrO_2_, Y_2_O_3_, MgO, MgF_2_, MgAl_2_O_4_ ↑temperature oxidation resistance (6× higher than untreated)	PDP in 3.5 wt.% NaCl (E_corr_; i_corr_) Untreated: −1.4383 V; 4.7134 × 10^−5^ A/cm^2^ PEO: −1.4326 V; 1.8598 × 10^−7^ A/cm^2^	[191]
	borate potassium acid phthalate (KAP) 0–6.0 g/L	AZ91D NaOH, Na_2_B_4_O_7_ AC, 120 V, 50 Hz 30 °C, 3 min	↓thickness 39.18 µm (0 g/L) to 23.07 µm (6 g/L) MgO ↓current density ↓vigorous sparking ↓gas evolution ↓pores and cracks ↑compactness ↑smoothness	PDP in 3.5 wt.% NaCl (E_corr_; i_corr_) 0 g/L: −1.502 V; 3.092 × 10^−6^ A/cm^2^ 4 g/L: −1.372 V, 2.001 × 10^−7^ A/cm^2^	[192]
	calcium glycerophosphate (CaGly)	AZ31B Na_2_SiO_3_, Na_3_PO_4_, KOH, KF, Na_2_EDTA, DC: 100 mA cm^−2^, 200 V, 20 °C, 45 s	1 µm, MgO tested with and without 25 µm-thick inhibitor-free epoxy primer	EIS in 0.5 wt.% NaCl (|Z|_10mHz_), 30 min/24 h Stand-alone: 10^6^ Ωcm^2^/2 × 10 Ωcm^2^ NSST, 7 d With primer: creepage 1.9 ± 0.28, ASTM D1654 score 7.	[193]
	Nd(NO_3_)_3_ 20, 40, 60, 80, 100 mM as a pre-treatment	KBM10 Na_2_SiO_3_, KOH, Al_2_O_3_, NaF, AC, 350 V, 400 Hz, 15 min	60 mM: ~23 µm Nd(NO_3_)_3_ precipitates in the alkaline electrolyte → used as a pre-treatment.	PDP in 3.5 wt.% NaCl (E_corr_; i_corr_) Bare alloy: −1.512 V; 1.80 × 10^−4^ A/cm^2^ 60 mM: −1.380 V; 8.02 × 10^−7^ A/cm^2^	[194]
	nickel acetate (NiAc_2_) 0.5 g L^−1^	AZ63B Na_2_SiO_3_, Na_2_B_4_O, NaOH, TEA DC pulsed: 3 A dm^−2^, 400 Hz, duty cycle 50%, 10–30 °C, 570 s	~35 µm MgO, MgSiO_3_, Mg_2_SiO_4_, Al_2_O_3_, SiO_2_, Ni_2_SiO_4_	NSST 500 h, corroded area PEO: 26.3% PEO-Ni: 9.6%	[195]
Organic	ethylene glycol oligomers EG10 g/L PEG_400_ 10 g/L PEG_1000_ 10 g/L PEG_4000_ 10 g/L	AZ31B NaOH, NaSiO_3_, Na_2_B_4_O_7_ C_6_H_5_O_7_Na_3_ DC 10 mA cm^−2^ 10 min	Thickness undisclosed V_end_ < 130 V ↑breakdown potential (EG, PEG_400_, and PEG_1000_) MgO, MgSiO_3_, Mg_2_SiO_4_ ↓roughness ↑compactness	PDP in 3.5 wt.% NaCl (E_corr_; i_corr_) corrosion resistance PEG_400_ < PEG_4000_ < PEG_1000_ Untreated: −1.478 V; 1.949 × 10^−4^ A/cm^2^ 0 g/L: −1.422 V; 3.673 × 10^−6^ A cm^−2^ PEG_1000_: −1.222 V; 1.232 × 10^−7^ A/cm^2^	[186]
	EDTA 0.5 g/L	Mg—5 mass % Li Na_2_SiO_3_, Na3PO_4_, NaF, NaOH constant current density 2 A dm^−2^, pulse frequency of 300 Hz duty cycle 45% 5 min	↓thickness 13 µm (0 g/L) to 9.5 µm (0.5 g/L) V_end_ < 450 V MgO, Mg_2_SiO_4_ ↑uniformity	PDP in 3.5 wt.% NaCl (E_corr_; i_corr_) 0 g/L: −1.522 V; 1.37 × 10^−6^ A/cm^2^ 0.5 g/L: −1.535 V; 3.76 × 10^−8^ A/cm^2^	[196]
	dodecyl sodium sulfate 0.25 g/L, diphenylamine-4-sulfonic acid sodium, 0.25 g/L dodecyl phenyl sodium sulfonate 0.25 g/L	AZ31B Na_2_SiO_3_, KF, KOH, glycerol DC 100 mA cm^−2^, 10 min	Thickness undisclosed V_end_ < 500 V ↓V_breakdown_ by 16–18 V easier release of oxygen ↓porosity 7.5% to 0.8% for dodecyl phenyl sodium sulfonate ↑quality	-	[66]
	glycerol 0–6 mL/L	AZ91D Na_2_SiO_3_, NaOH, Na_2_EDTA pulse-reverse voltage positive 400 V/negative120 V, frequency 100 Hz 15 min	↓thickness: 111 µm (0 mL/L) to 64 µm (6 mL/L) MgO, Mg_2_SiO_4_ ↓interfacial tension ↑number of small size intensive sparks ↑smoothness ↓pore size and cracks	PDP in 3.5 wt.% NaCl (E_corr_; i_corr_) 0 mL/L: −1.512 V; 6.16 × 10^−5^ A/cm^2^ 2 mL/L: −1.483 V; 5.06 × 10^−5^ A/cm^2^	[187]
	tannic acid 4 g/L	AZ91 NaOH, optional Na_2_SiO_3_ Constant current density 40 mA/cm^2^ the unipolar positive pulse, frequency 2000 Hz, duty cycle 20%, 15–40 °C, 3 min	thickness: 4.4 μm (NaOH + Na_2_SiO_3_), discontinuous (4 g/L + NaOH) 6.6 μm (4 g/L + NaOH + Na_2_SiO_3_) V_end_ 288 V (0 g/L), 171 V (4 g/L), 338 V (4 g/L + Na_2_SiO_3_) insoluble magnesium-tannate complex presence ↑pore uniformity effect on coating color	PDP in 3.5 wt.% NaCl (E_corr_; i_corr_) Untreated: −1.576 V; 1.584 × 10^−4^ A/cm^2^ NaOH, Na_2_SiO_3_: −1.14 V; 6.125 × 10^−7^ A/cm^2^ NaOH, 4 g/L: −1.513 V; 1.226 × 10^−5^ A/cm^2^ NaOH,4 g/L, Na_2_SiO_3_: −1.349 V; 1.385 × 10^−7^ A/cm^2^	[197]
	benzotriazole (BTA) 3, 5, 10 g/L	AZ31 KOH, Na_2_SiO_3_, Na_2_B_4_O_7_, Na_2_CO_3_, Na_2_SiO_3_ DC 1.5 A dm^−2^_23_ 10 min	thickness: 12 (0 g/L) to 20 µM (5 g/L) ↑V_end_ 65 V to 115 V (5 g/L) Mg_2_SiO_4_, MgO best properties for 5 g/L BTA ↑uniformity, compactness formation of BTA adsorption layer on Mg alloy substrate	PDP_30 min_ in 0.005 M NaCl (E_corr_; i_corr_) Untreated: −1.517 V; 1.272 × 10^−5^ A/cm^2^ 0 g/L: −1.486 V; 5.134 × 10^−7^ A/cm^2^ 5 g/L: −1.303 V; 2.193 × 10^−7^ A/cm^2^	[185]
	8-hydroxyquinoline (HQ) 2, 5, 8 g/L	AZ91 NaOH, Na_2_SiO_3_ Constant current density 40 mA cm^−2^, frequency 2000 Hz, duty cycle 20%, 15–40 °C, 3 min	↑thickness: 5.0 µm (0 g/L) to 6, 7.5, 6 µm (2,5, 8 g/L) V_end_< 316 V (5 g/L) MgO, Mg_2_SiO_4_, MgAl_2_O_4_, insoluble Mg(HQ)_2_ ↓conductivity ↓pore size changing coating color	PDP in 3.5 wt.% NaCl (E_corr_; i_corr_) 0 g/L: −1.528 V; 4 × 10^−5^ A/cm^2^ 2 g/L: −1.480 V; 2.2 × 10^−6^ A/cm^2^ 5 g/L: −1.457 V, 3.2 × 10^−6^ A/cm^2^ 8 g/L: −1.474 V; 3.6 × 10^−6^ A/cm^2^	[198]
	ethylene–glycol 10–70 wt%	Magnesium (99.95%) KOH, Na_2_SiO_3_ DC 50 Am^−2^ 20 °C, 10 min	Thickness undisclosed ↑V_end_ < 200 V anodic film consisted of two layers (heterogeneous porous layer and a barrier layer)	0.5 wt.% NaCl solution immersion time >10 min best polarization resistance for 20 wt% ethylene–glycol solution, 10-fold greater than HAE method	[126]
	3-aminopropyltrimethoxysilane (APTMS) 0.02 mol/L	AZ31B Na_2_SiO_3_, NaOH, NaF DC pulsed 5 A dm^−2^, 500 Hz, duty cycle 10%, <30 °C, 5 min	14 µm MgO, Mg_2_SiO_4_, metallo-siloxane bonds (Mg-O-Si) in the coating	PDP in 3.5 wt.% NaCl (E_corr_, i_corr_) 0 mol/L: −1.457 V; 8.32 × 10^−7^ A/cm^2^ 0.02 mol/L: −1.265 V; 1.23 × 10^−7^ A/cm^2^	[199]
	HCONH_2_ 245 mL/L	AZ91D (1) PENC pretreatment: HCONH_2_, NaOH DC cathodic: 180 V 30 min (2) PEO: Na_2_SiO_3_, KF, NaOH AC: 400 V, 700 Hz, duty cycle 20%, 10 min	PENC: ~15 µm oxide and diffusion layer, MgO, Al_2_O_3_, MgC_2_ Mg_3_N_2._ PENC + PEO: ~20 µm dense oxide layer, MgO, Al_2_O_3_, MgF_2_, Mg_2_SiO_4_, SiC, Si_3_N_4_, MgC_2_, Mg_3_N_2_	PDP in 3.5 wt.% NaCl (i_corr_) PENC + PEO: 1.92 × 10^−8^ A/cm^2^, two and one orders of magnitude greater than for PENC and PEO. EIS in 3.5 wt.% NaCl (|Z|_10mHz_), 30 min/72 h PENC + PEO: 10^7^ Ω cm^2^/10^6^ Ω cm^2^ PEO: 3 × 10^6^ Ω cm^2^/3 × 10^3^ Ω cm^2^ PENC: 1.7 × 10^4^ Ω cm^2^/1.7 × 10^2^ Ω cm^2^	[200]

**Table 5:** Effect of in situ particle additives in PEO electrolytes on the structural and corrosive properties of the obtained coating.

Particles	Alloy Electrolyte Treatment Conditions	Thickness Phases Incorporation	Corrosion Data	Ref.
9 g/L m- and t- ZrO_2_, 200–400 nm	AZ91 KOH, KF, K_4_P_2_O_7_ AC 50 mA/cm^2^, 7 min	~10 µm MgO, t-ZrO_2_, Mg_3_(PO_4_)_2_ Inert	PDP in 3.5 wt.% NaCl (E_corr_; i_corr_) Untreated: −1.56 V; 2.46 × 10^−5^ A/cm^2^ 0 g/L: −1.40 V; 7.3 × 10^− 7^ A/cm^2^ 9 g/L: −1.31 V; 7 × 10^− 8^ A/cm^2^ ASTM B117 48 h ↓pits 120 h ≈ pits (saturation of pitting corrosion)	[213]
5 vol.% ZrO_2_ sol	AZ91D Na_2_SiO_3_, KOH DC_pulsed_10 A/dm^2^, 20 min, 200 Hz, 15% duty cycle	36 to 40 μm (5 vol% ZrO_2_) MgO, Mg_2_SiO_4_, Mg_2_Zr_5_O_12_ Reactive	PDP_10min_ in 3.5 wt.% NaCl (E_corr_; i_corr_) Untreated: −1.55 V; 4.378 × 10^−5^ A/cm^2^ 0 vol.%: −1.50 V; 5.3 × 10^−7^ A/cm^2^ 5 vol.%: −1.22 V;1.4 × 10^−8^ A/cm^2^	[212]
5 g/L n-SiO_2_~12 nm µ-SiO_2_ 1–5 μm	AM50 Na_3_PO_4_, KOH DC_pulsed_450 V, 10 min, 250 Hz, 10% duty cycle	↓45 ± 5, 33 ± 3 µm (µ-SiO_2_), 25± 4 (n-SiO_2_) Amorphous coating (n-SiO2), Amorphous, MgO, SiO_2_ (µ-SiO_2_) Reactive (n-SiO_2_) Inert (µ-SiO_2_)	PDP_30 min_ in 0.5 wt.% NaCl (E_corr_; i_corr_) 0 g/L: −1.59 V; (1.2 ± 0.2) × 10^−7^ A cm^−2^ 5 g/L n-SiO_2_: −1.55 V; (2.4 ± 1.6) × 10^−7^ A cm^−2^ 5 g/L µ-SiO_2_: −1.57 V; (1.9 ± 0.7) × 10^−7^ A cm^−2^	[231]
1 vol.% silica sol	Mg-Li alloy Na_2_SiO_3_, NaOH DC_pulsed_5 A/dm^2^, 10 min, 2000 Hz, 15% duty cycle	? µm MgO, SiO_2_, Mg_2_SiO_4_ Partly reactive	PDP_5 min_ in 3.5 wt.% NaCl (E_corr_; i_corr_) 0 vol.%: 6.3 × 10^−7^ A/cm^2^ 1 vol.%: 1.0 × 10^−7^ A/cm^2^	[230]
5 g/L clay 12 μm	AM50 KOH, Na_3_PO_4_ DC_pulsed_450 V, 10 min, i_limit_ 0.3 A/cm^2^, t_on_:t_off_ = 2 ms:18 ms, 10 ±2 °C (1) h = high, m = medium, s = standard concentration (2) h = hydroxide, p = phosphate	hh- 40 ± 5 μm, MgO, Mg_3_(PO_4_)_2_, Mg_2_SiO_4_ mh- 50 ± 8 μm MgO, Mg_3_(PO_4_)_2_, Mg_2_SiO_4_ mp- 43 ± 7 μm amorphous phase hp- 67 ± 6 μm amorphous phase s- 15 ± 5 μm MgO Reactive	PDP_30 min_ in 0.5 wt.% NaCl (E_corr_; i_corr_) AM50: −1.48 V; (3.5 ± 1.1) × 10^−6^ A/cm^2^ hh-PEO: −1.54 V;(5.8 ± 0.8) × 10^−8^ A/cm^2^ mh-PEO−1.56 V; (2.3 ± 1.4) × 10^−8^ A/cm^2^ s-PEO−1.48 V; (6.3 ± 10.7) × 10^−7^ A/cm^2^ mp-PEO−1.56 V; (6.3 ± 1.7) × 10^−8^ A/cm^2^ hp-PEO−1.57 V; (1.9 ± 1.6) × 10^−7^ A/cm^2^	[116]
2 vol.% alumina sol	AZ91D NaAlO_2_, KOH DC_pulsed_15 mA/cm^2^, 25 min	? µm MgO, MgAl_2_O_4_ Partly reactive	PDP_30 min_ in 3.5 wt.% NaCl (E_corr_; i_corr_) Untreated: −1.51 V; 2.352 × 10^−6^ A/cm^2^ 0 vol%: −1.48 V; 1.6 × 10^−6^ A/cm^2^ 2 vol%: −1.38 V; 2.6 × 10^−8^ A/cm^2^	[228]
10 g/L Al_2_O_3_ 500 nm	AZ31 Na_2_SiO_3_, NaOH, phytic acid, ethylene diamine tetra acetic acid, polymeric surfactant DC 15 mA/cm^2^, 20 min	↑15–17 to 16–20 μm MgO, Mg_2_SiO_4_, Al_2_O_3_ Inert	PDP_10 min_ in 3.5 wt.% NaCl (E_corr_; i_corr_) Untreated: −1.48 V; 5.088 × 10^−5^ A/cm^2^ 0 g/L: −1.19 V; 8.4 × 10^−7^ A/cm^2^ 10 g/L: −1.14 V; 3.8 × 10^−7^ A/cm^2^	[227]
4 vol.% TiO_2_	AM60B Na_3_PO_4_, KOH DC_bipolar_6 A/dm^2^, 26–30 min, 150 Hz, 37.5% duty cycle	~37 µm MgO, MgAl_2_O_4_, rutile, anatase Inert	PDP_10 min_ in 3.5 wt.% NaCl (E_corr_; i_corr_) Untreated: −1.62 V; 5.2 × 10^−5^ A/cm^2^ 0 vol.%: −1.58 V; 4.2 × 10^−6^ A/cm^2^ 4 vol.%: −1.51 V; 4.3 × 10^−8^ A/cm^2^	[226]
2, 4, 6 g/L TiO_2_ nanoparticles	AZ91D (NaPO_3_)_6_, NaOH DC_pulsed_3 A/dm^2^, 15 min, 200 Hz, 15% duty ratio	29 μm (0 g/L), 35 µm (4 g/L) MgO, MgAl_2_O_4_, Mg_3_(PO_4_)_2_, rutile, anatase, Mg_2_TiO_4_ Partly reactive	PDP_20min_ in 3.5 wt.% NaCl (E_corr_; i_corr_) Untreated: −1.55 V; 4.4 × 10^−5^ A/cm^2^ 0 g/L: −1.51 V; 6.9 × 10^−7^ A/cm^2^ 4 g/L: −1.49 V; 1.9 × 10^−8^ A/cm^2^ 6 g/L: −1.43 V; 3.8 × 10^−8^ A/cm^2^	[225]
10, 20, 30 g/L CeO_2_ <5 μm	AZ31 Na_2_SiO_3_, KF DC_pulsed_ 5 A/dm^2^, 10 min, 100 Hz, 6% duty cycle	~10 µm Mg_2_SiO_4_, CeO_2_ Inert	PDP_10min_ in 3.5 wt.% NaCl (E_corr_; i_corr_) Untreated: −1.55 V; 4.5 × 10^− 5^ A/cm^2^ 0 g/L: −1.54 V; 8.6 × 10^−6^ A/cm^2^ 10 g/L: −1.53 V; 1.0 × 10^−6^ A/cm^2^ 20 g/L: −1.52 V; 2 × 10^−7^ A/cm^2^ 30 g/L: −1.45 V; 4 × 10^−8^ A/cm^2^	[220]
1 g/L CeO_2_	AZ31 NaOH 10 V (anodizing below breakdown) 30 min	? µm Mg(OH)_2_, CeO_2_ Inert	PDP in 17 mM NaCl-0.1 M Na_2_SO_4_ (E_corr_; i_corr_) Untreated: −1.46 V 0 g/L: −1.35 V 1 g/L: −1.17 V	[219]
0, 1, 3, 5 g/L Y_2_O_3_ 40 nm	AZ91 Na_2_SiO_3_, KOH, Na_5_P_3_O_10_, glycerol, Y_2_O_3_ Bipolar pulsed: +450/−30 V, 800 Hz, duty cycle 10%, duty ratio 1:1, 20 min	0 g/L: ~57 µm 3 g/L: ~53 µm 5 g/L: ~37 µm MgO, Mg_2_SiO_4_, Y_2_O_3_ Inert	PDP_30 min_ in 3.5 wt.% NaCl (E_corr_; i_corr_) Untreated: −1.57 V; 1.78 × 10^− 4^ A/cm^2^ 0 g/L: −1.43 V; 1.38 × 10^−6^ A/cm^2^ 3 g/L: −1.40 V; 1.62 × 10^−7^ A/cm^2^ 5 g/L: −1.42 V; 2.29 × 10^−7^ A/cm^2^	[223]
0, 2, 4, 6, 8 g/L Sb_2_O_3_ 150 nm	DC_pulsed_: 4 A dm^−2^, 100 Hz, duty cycle 30%. <30 °C, 30 min.	0 g/L: ~11 µm 2 g/L: ~14 µm 4 g/L: ~18 µm, 6 g/L: ~13 µm, 8 g/L: ~12 µm MgO, Mg_2_SiO_4_, and SiO_2_, ~0.9 at.% Sb max.	PDP in 3.5 wt.% NaCl (E_corr_; i_corr_) 0 g/L: −1.475 V; 1.003 × 10^−8^ A/cm^2^ 2 g/L: −1.520 V; 4.163 × 10^−9^ A/cm^2^ 4 g/L: −1.550 V; 1.878 × 10^−9^ A/cm^2^, 6 g/L: −1.531 V; 1.154 × 10^−8^ A/cm^2^, 8 g/L: −1.415 V; 1.355 × 10^−8^ mm/year,	[224]
2 g/L SiC 50 nm	AZ31 Na_2_SiO_3_·(NaPO_3_)_6_ DC_bipolar_, 1000 Hz, 20 min, 20% duty cycle, 40 °C Higher positive/negative current densities (HC) ~0.22/0.09 A cm^−2^ Lower positive/negative current densities (LC) ~0.13/0.03 A cm^−2^	HC-SiC 126 μm, HC 121 μm, LC-SiC 88 μm, LC 76 μm Mg_2_SiO_4_, MgO, SiC Inert	PDPin 5 wt.% NaCl (i_corr_) Untreated: 1.56 × 10^−4^ A/cm^2^ Coatings: 2.46 × 10^−6^–9.25 × 10^−7^ A/cm^2^(PEO-HC-SiC-the best result)	[235]
5 g/L Si_3_N_4_ 0.02 μm 0.1–0.8 μm 1–5 μm	AM50 Na_3_PO_4_, KOH DC_pulsed_ 450 V, 10 min, 50 Hz, 10% duty cycle	(0.02 µm) 40 μm (0.1–0.8 µm) 25 μm (1–5 µm) 10 µm MgO, Si_3_N_4_, Mg_3_(PO_4_)_2_ Inert	PDP_30min_ in 0.5 wt.% NaCl (E_corr_; i_corr_) Untreated: −1.452 V; 3.5 × 10^−5^ A/cm^2^ 0.02 μm: −1.509 V; 9 × 10^−8^ A/cm^2^ 0.1–0.8 μm: −1.559 V; 1.9 × 10^−7^ A/cm^2^ 1–5 μm: −1.575 V; 3.5 × 10^−7^ A/cm^2^	[241]
1, 2, 3, 4 g/L Si_3_N_4_ 50 nm	AZ31 K_3_PO_4_, NaAlO_2_ dispersant: alcohol and surfactant SDS DC_pulsed_83.3 mA/cm^2^, 475 V_max_, 10 min, 1000 Hz, 50% duty cycle	13.1 μm (0 g/L) 17.8 μm (3 g/L), 16.8 μm (4 g/L) MgAl_2_O_4_, MgO, Mg_2_SiO_4_ Reactive	PDP in 3.5 wt.% NaCl (E_corr_; i_corr_) Untreated: −1.5 V; 1.56 × 10^−5^ A/cm^2^ 0 g/L: −0.10 V; 2.96 × 10^−6^ A/cm^2^ 1 g/L: −0.17 V; 2.11 × 10^−6^ A/cm^2^ 2 g/L: −0.19 V; 1.95 × 10^−6^ A/cm^2^ 3 g/L: −0.22 V; 4.23 × 10^−6^ A/cm^2^ 4 g/l: −0.22 V; 6.20 × 10^−6^ A/cm^2^	[239]
1,2,3,4 g/L TiN 20 nm	MA8 NaF, Na_2_SiO_3_, sodium dodecylsulfate 1 step: DC_bipolar_ 0.5 A cm^−2^, 300 Hz, 50% duty cycle, constant potential cathodic pulse (−30 V), 200 s, reached voltage 300 V 2 step:voltage dynamically reduced to 200 V, cathodic pulse reduced to(−10 V), 600 s	~20 µm MgO, Mg_2_SiO_4_, TiN Inert	PDPin 3 wt.% NaCl (E_corr_; i_corr_) 0 g/L: −1.37 V; 1.2 × 10^−7^ A/cm^2^ 1 g/L: −1.44 V: 1.4 × 10^−7^ A/cm^2^ 2 g/L: −1.45 V; 1.6 × 10^−7^ A/cm^2^ 3 g/L: −1.47 V; 1.8 × 10^−7^ A/cm^2^ 4 g/L: −1.50 V, 7.9 × 10^−7^ A/cm^2^	[240]
1, 2, 3 g/L graphene oxide	AZ31 Na_2_HPO_4_, NaF, sodium citrate, glycerol, sodium dodecyl sulfate 1 step DC_pulsed_ 100 mA/cm^2^, 500 Hz, 10% duty cycle, 20–30 °C, 1 min 2 step graphene addition, constant voltage(400 V), 9 min	PEO 40.7 μm 1 g/L 35.9 μm 2 g/L 34.3 μm 3 g/L 36.7 μm MgO	PDP in 3.5 wt.% NaCl (E_corr_; i_corr_) Untreated: −1.48 V; 1.08 × 10^−5^ A/cm^2^ 0 g/L: −1.49 V; 1.24 × 10^−7^ A/cm^2^ 1 g/L: −1.47 V; 9.8 × 10^−8^ A/cm^2^ 2 g/L: −1.44 V; 3.3 × 10^−8^ A/cm^2^ 3 g/L: −1.41 V; 7.1 × 10^−8^ A/cm^2^	[242]
Self-healing 10 g/L halloysite nanotubes (HNT) or benzotriazole loaded HNT (BTA-HNT) 1–15 μm length 10–100 nm inner diameter	AM50 Na_2_SiO_3_, KOH, NaF DC_pulsed_ 40 mA/cm^2^, 10 min, 100 Hz, 10% duty cycle	0 g/L 30.1 µm HNT 29.5 µm BTA-HNT-P 36.2 µm Amorphous, Mg_2_SiO_4_, halloysite Inert	Potentiodynamic polarization is unsuitable because of dynamic processes on the polarized surface and in the electrolyte. BTA-HNT-PEO coating showed the smallest variation in |Z| during the 12 h of immersion, indicating the most stable corrosion resistance and protection to the substrate.	[250]
Multiwalled carbon nanotubes 2.5, 5, 10 g/L	AZ31 Na_2_SiO_3_, KOH, KF DC 100 mA/cm^2^, 10 min	0 g/L 19.3 μm 2.5 g/L 14.2 μm 5 g/L 12.5 μm 10 g/L 8.0 μm MgO, Mg_2_SiO_4_ CNT oxidizes during PEO	PDP and EIS in 3.5 wt.% NaCl (E_corr_; i_corr_^;^ |Z|_10mHz_) 0 g/L: −1.49 V; 7.14 × 10^−6^ A/cm^2^; 10^4^ Ωcm^2^ 2.5 g/L: −1.45 V; 3.67 × 10^−6^ A/cm^2^ 5 g/L: −1.47 V; 1.43 × 10^−6^ A/cm^2^ 10 g/L: −1.38 V; 4.80 × 10^−7^ A/cm^2^; 10^5^ Ωcm^2^	[247]

## Data Availability

Data sharing not applicable.
